# Supplementary material for: Direct detection of an NH-π hydrogen bond in an intrinsically disordered peptide
Source: Nat Commun. 2025 Nov 20;16:10231. doi: 10.1038/s41467-025-66013-2 (PMC12635323; doi:10.1038/s41467-025-66013-2)
Supplement: Supplementary file 1 — Supplementary Information [file 41467_2025_66013_MOESM1_ESM.pdf]

Direct detection of an NH-π hydrogen bond in an intrinsically disordered peptide

Luigi Russo[1]†, Dipendu Dhar[2]†, Robin Backer[3], Om Prakash[4], Fatima Matroodi[5], Kerstin Overkamp[6], Karin Giller[6], Stefan Becker[6], Christian Griesinger[6], Dieter Willbold[3,7], Barbara Rossi[5,8], Mehdi D. Davari[2]*, Nasrollah Rezaei-Ghaleh[3,7,9]*

[1]Department of Environmental, Biological and Pharmaceutical Science and Technology, University of Campania-Luigi Vanvitelli; Via Vivaldi 43, I-81100 Caserta, Italy.

[2]Department of Bioorganic Chemistry, Leibniz Institute of Plant Biochemistry; Winberg 3, D-06120 Halle, Germany.

[3]Institute of Physical Biology, Heinrich Heine University Düsseldorf; Universitätsstraße 1, D-40225 Düsseldorf, Germany.

[4]Department of Physics, University of Pavia; Via Bassi 6, I-27100 Pavia, Italy.

[5]Elettra Sincrotrone Trieste; Strada Statale 14 km 163.5, Area Science Park, I-34149 Trieste, Italy.

[6]Department of NMR-based Structural Biology, Max Planck Institute for Multidisciplinary Sciences; Am Faßberg 11, D-37077 Göttingen, Germany.

[7]Institute of Biological Information Processing, IBI-7: Structural Biochemistry, Forschungszentrum Jülich; Wilhelm-Johnen-Straße, D-52428 Jülich, Germany.

[8]Department of Physics, University of Trento; Via Sommarive 14, I-38123 Povo Trento, Italy.

[9]Department of Molecular Medicine, University of Pavia; Via Ferrata 9, I-27100 Pavia, Italy.

† These authors contributed equally.

* Corresponding authors:

Dr. Mehdi D. Davari; E-Mail: Mehdi.Davari@ipb-halle.de

Dr. Nasrollah Rezaei-Ghaleh; E-Mail: Nasrollah.RezaieGhaleh@unipv.it

This pdf file includes:

Supplementary Figures 1-20, Supplementary Tables 1-5, Supplementary Note, and Supplementary References. The original gel image used for Supplementary Fig. 3c is included at the end.

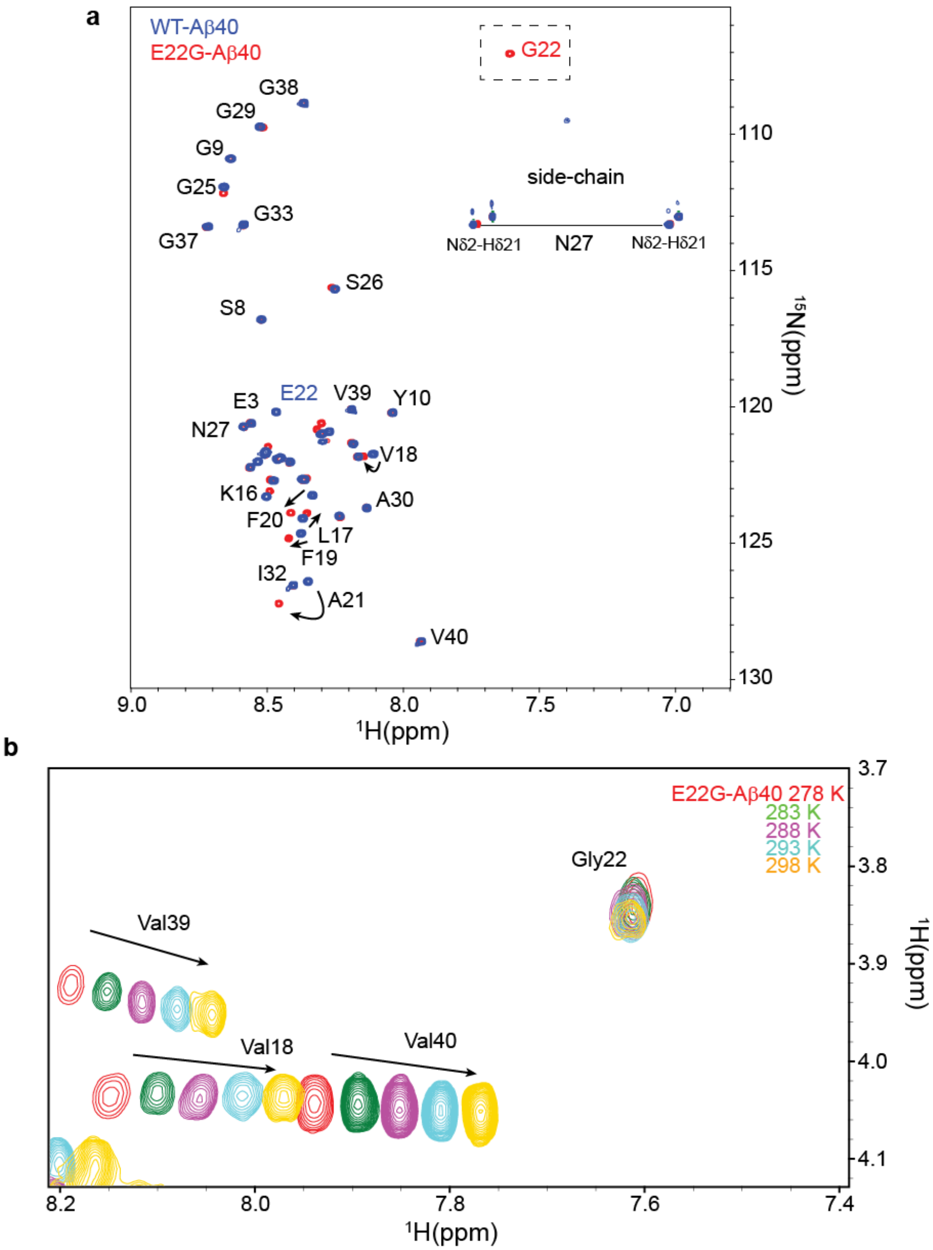

Supplementary Fig. 1. Upfield amide chemical shifts and the near-zero temperature coefficient of Gly22's amide proton chemical shift support the presence of an NH-π interaction. a Overlaid ^{15}N,^{1}H HSQC spectra of wild-type (WT) and E22G-Aβ40, showing the upfield chemical shifts of Gly22. Peak displacements observed for residues close to the mutation site are also highlighted. **b** ^{1}H,^{1}H TOCSY spectrum of the E22G-Aβ40 acquired at different temperatures in the 278-298 K range, showing the upfield H^{N} chemical shift of Gly22 and its near zero temperature coefficient.

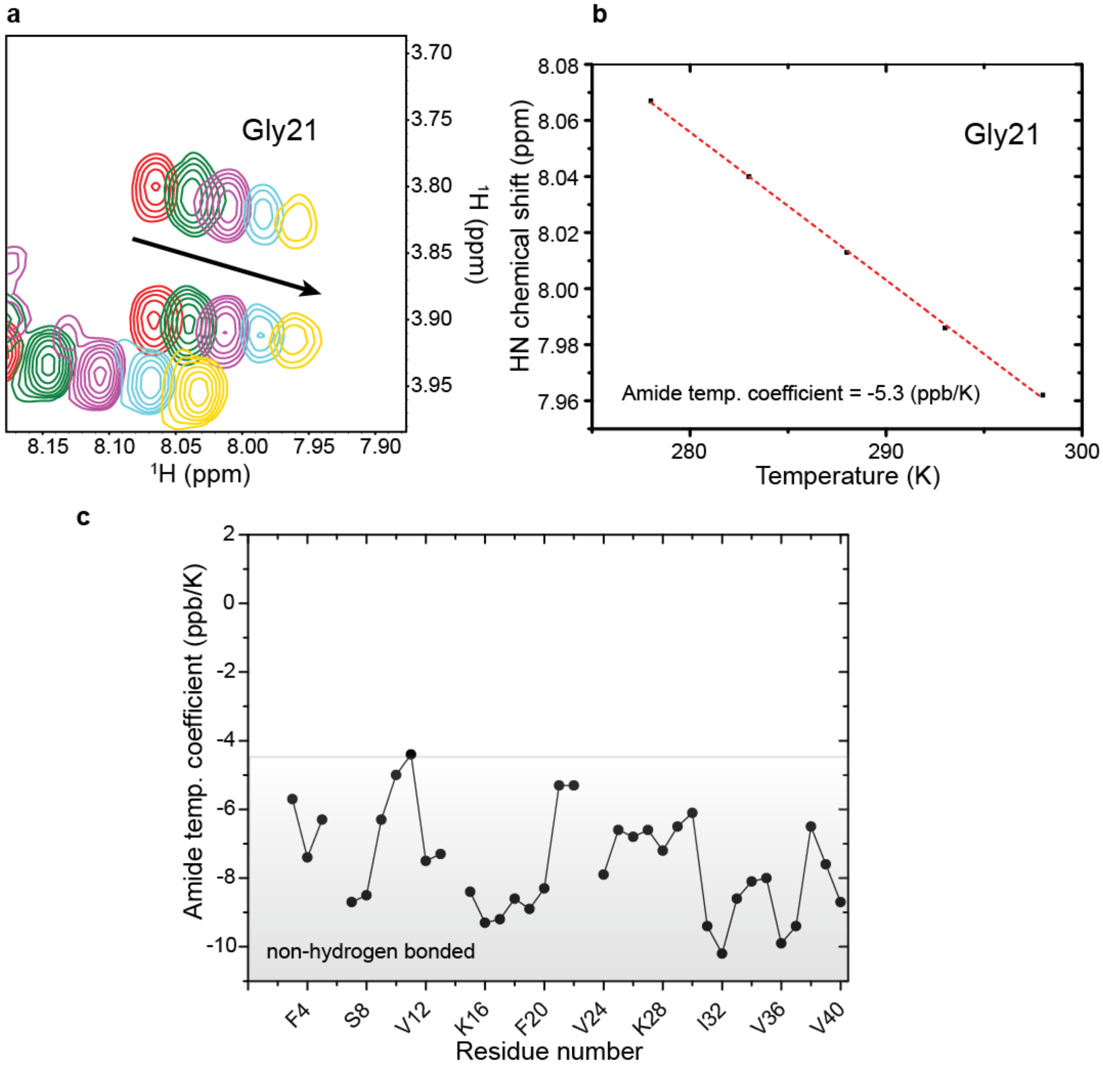

Supplementary Fig. 2. A21G-Aβ40 do not show the characteristic signature of an NH-π interaction engaging Gly21's amide group. a Portion of ^{1}H-^{1}H TOCSY spectrum of A21G-Aβ40 as a function of temperature, showing the lack of upfield chemical shift for Gly21's amide proton and its large sensitivity to temperature changes. **b** Plot reporting the temperature dependence of amide proton chemical shifts for Gly21. **c** Residue-specific temperature coefficients of HN chemical shifts for A21G-Aβ40, derived from ^{1}H,^{1}H TOCSY spectra. Similar to other residues, Gly21 exhibits a large negative temperature coefficient, characteristic of residues located in intrinsically disordered proteins.

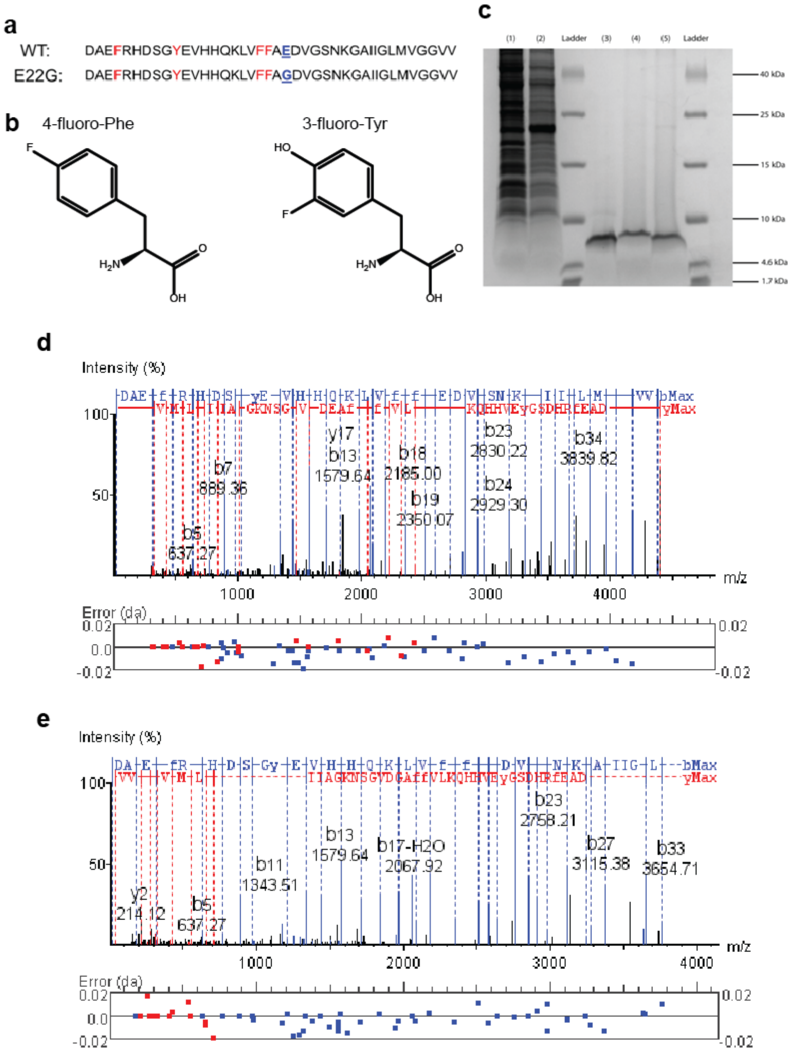

Supplementary Fig. 3. Recombinant production of fluorine-labeled Aβ40. a Aminoacid sequence of wild-type (WT) and E22G-Aβ40 in single letter code. The fluorinated residues Phe (F) and Tyr (Y) are highlighted in red and the mutated residue in blue. **b** 2D chemical structure of fluorinated Phe and Tyr aminoacids. **c** Tris/Tricine-SDS-PAGE of recombinantly produced Aβ40. (1) *E. coli* DL39(DE3) cells transformed with the E22G construct, cultivated in 2YT medium; (2) *E. coli* DL39(DE3) cells after induced expression of the E22G construct in defined medium; (3) recombinant fluoro-Phe, fluoro-Tyr-Aβ40 WT; (4) recombinant fluoro-Phe, fluoro-Tyr-Aβ40 E22G; (5) synthetic Aβ40 WT (Bachem #4014442); used protein ladder: Spectra™ Multicolor Low Range (Thermo #26628); Coomassie stained. **d,e** Tandem mass spectra of recombinantly produced F-Phe4, F-Tyr10, F-Phe19, F-Phe20-Aβ40 (main species in the non-fragmented mass spectra) with sequence analysis of the detected b-fragments (N- to C-term.) and y-fragments (C- to N-term.), confirming the peptide identities for both, WT (**d**) and E22G (**e**) peptides. Fluorinated residues are indicated in lowercase letters (f for Phe, y for Tyr).

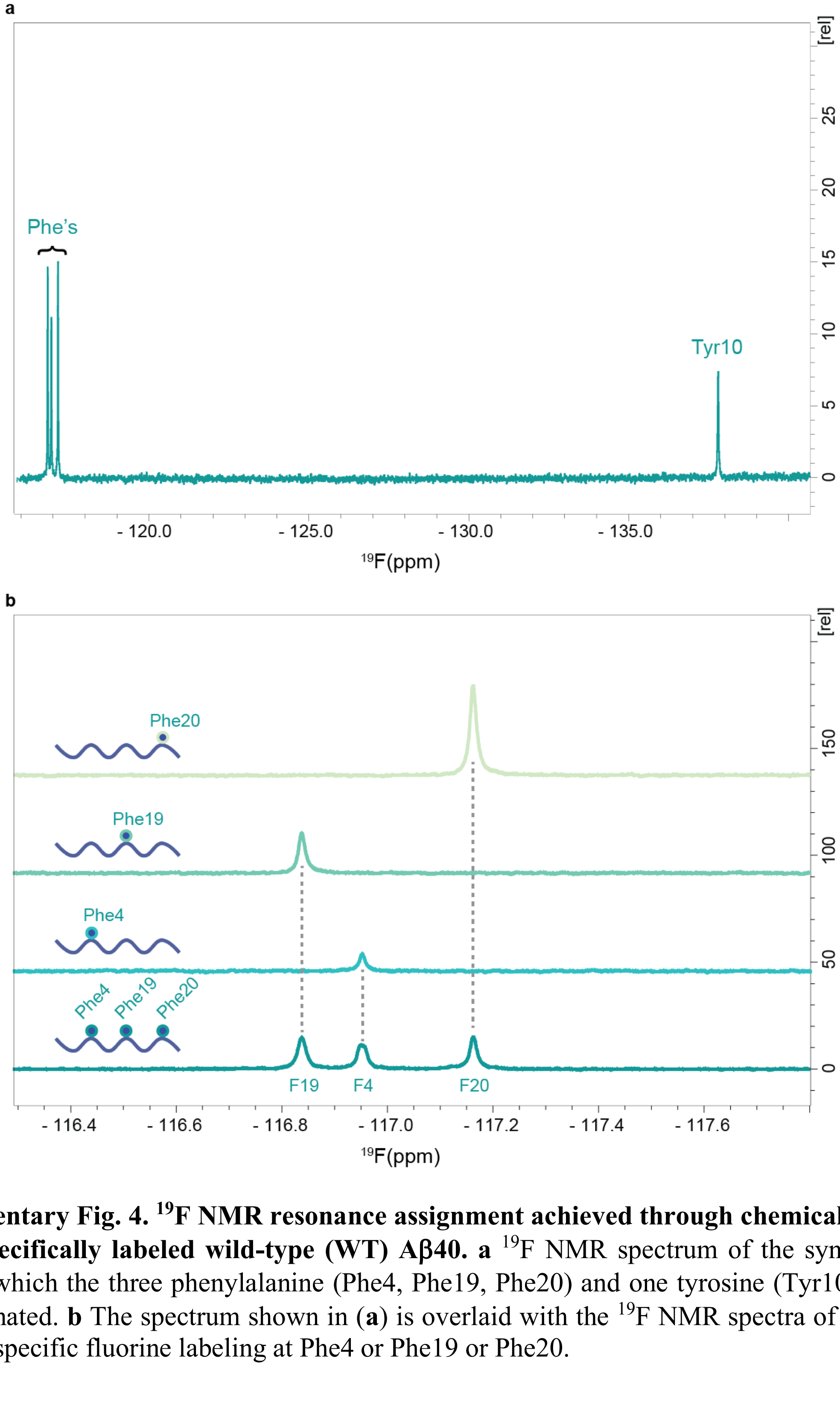

Supplementary Fig. 4. ^{19}F NMR resonance assignment achieved through chemical synthesis of site-specifically labeled wild-type (WT) Aβ40. a ^{19}F NMR spectrum of the synthetic WT Aβ40 in which the three phenylalanine (Phe4, Phe19, Phe20) and one tyrosine (Tyr10) residues are fluorinated. **b** The spectrum shown in (**a**) is overlaid with the ^{19}F NMR spectra of WT Aβ40 with site-specific fluorine labeling at Phe4 or Phe19 or Phe20.

a

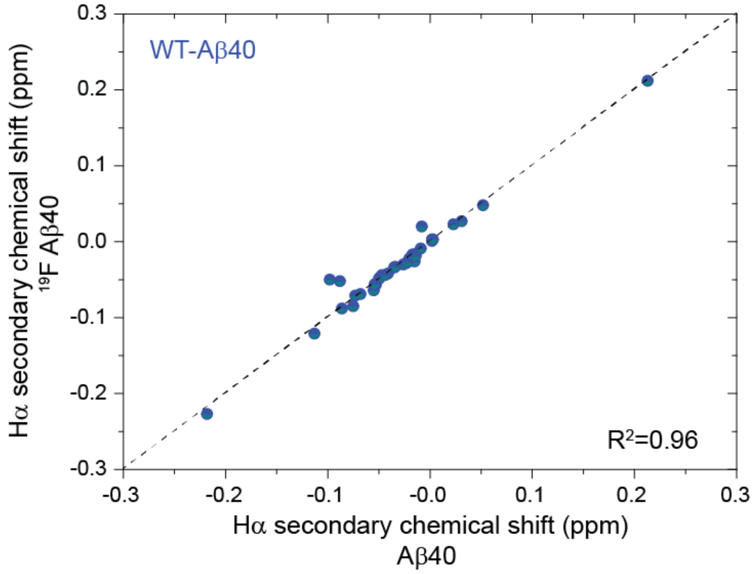

b

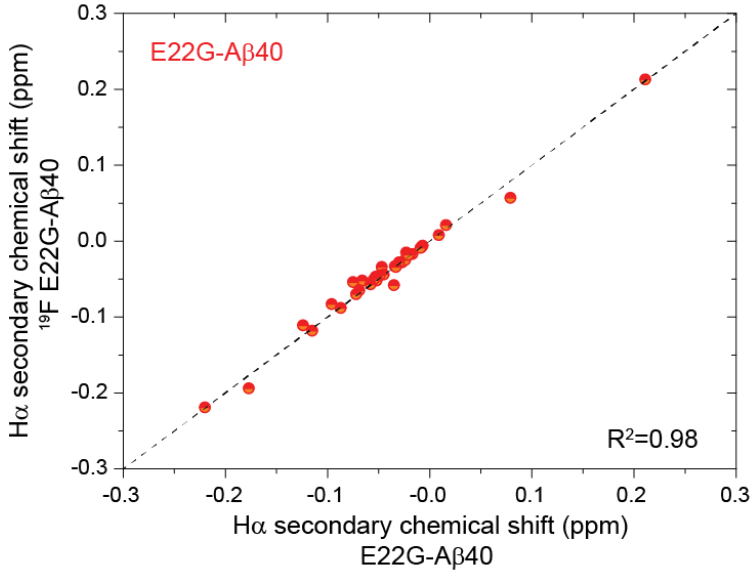

Supplementary Fig. 5. Effect of fluorine labeling of aromatic residues on the backbone conformation of wild-type (WT) and E22G Aβ40. a,b Comparison of Hα secondary chemical shifts does not show a significant alteration of backbone conformation in the WT (**a**) and E22G Aβ40 (**b**) peptides.

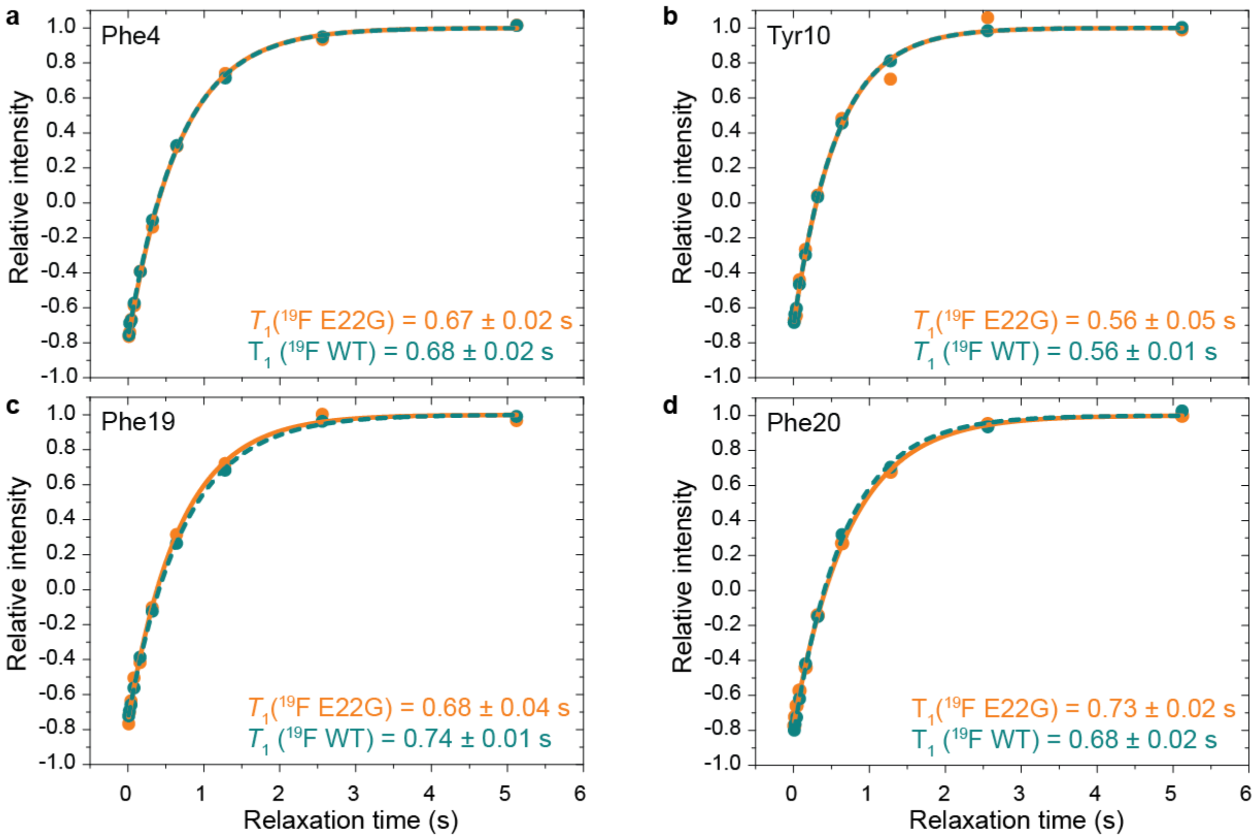

Supplementary Fig. 6. Dynamics of aromatic rings in the wild-type (WT) and E22G-Aβ40 peptides, probed through ^{19}F relaxation. a-d, ^{19}F longitudinal spin-lattice (T_1) relaxation times of Phe4 (**a**), Tyr10 (**b**), Phe19 (**c**) and Phe20 (**d**) in the WT and E22G peptides, obtained through inversion-recovery experiments, are shown. While residues Phe4 and Tyr10 do not show any significant change in the mutated peptide, residues Phe19 and Phe20 undergo respectively a small decrease or increase, reflecting their respective increase or decrease in nanosecond dynamics (see Supplementary Note).

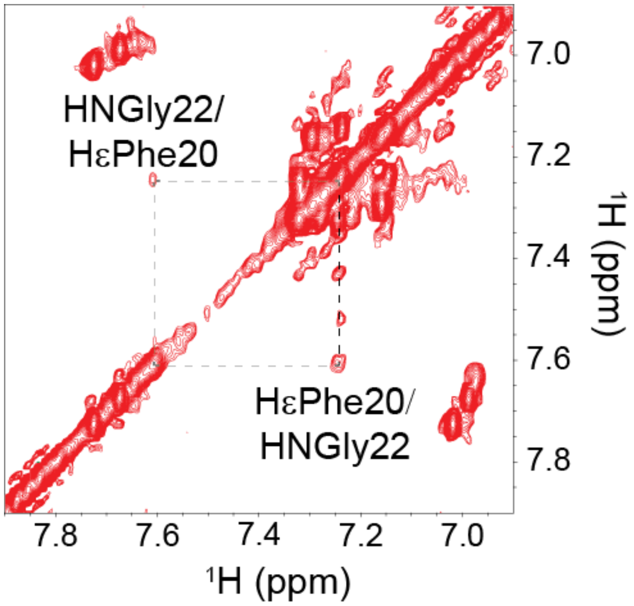

Supplementary Fig. 7. Spatial proximity of amide proton of Gly22 and aromatic protons of Phe20. A region of the 2D ^{1}H-^{1}H NOESY spectrum of E22G-Aβ40 showing through-space correlation between the amide proton (HN) of Gly22 and aromatic Hε protons of Phe20, in line with their involvement in the suggested NH-π interaction.

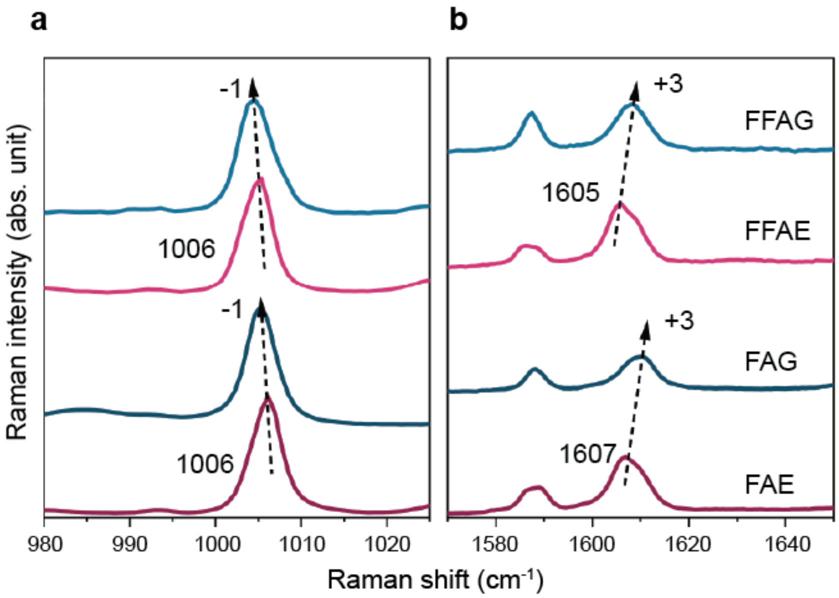

Supplementary Fig. 8. Comparison between the Raman spectra of truncated WT (FFAE and FAE) and E22G-Aβ (FFAG and FAG) peptides. a,b, The Raman bands of the mutated peptide associated with the vibrational modes ν_{12} (**a**) and ν_{8a} (**b**) of Phe rings show red and blue shifts, respectively, in line with the presence of an NH-π interaction in them (see Supplementary Fig. 10).

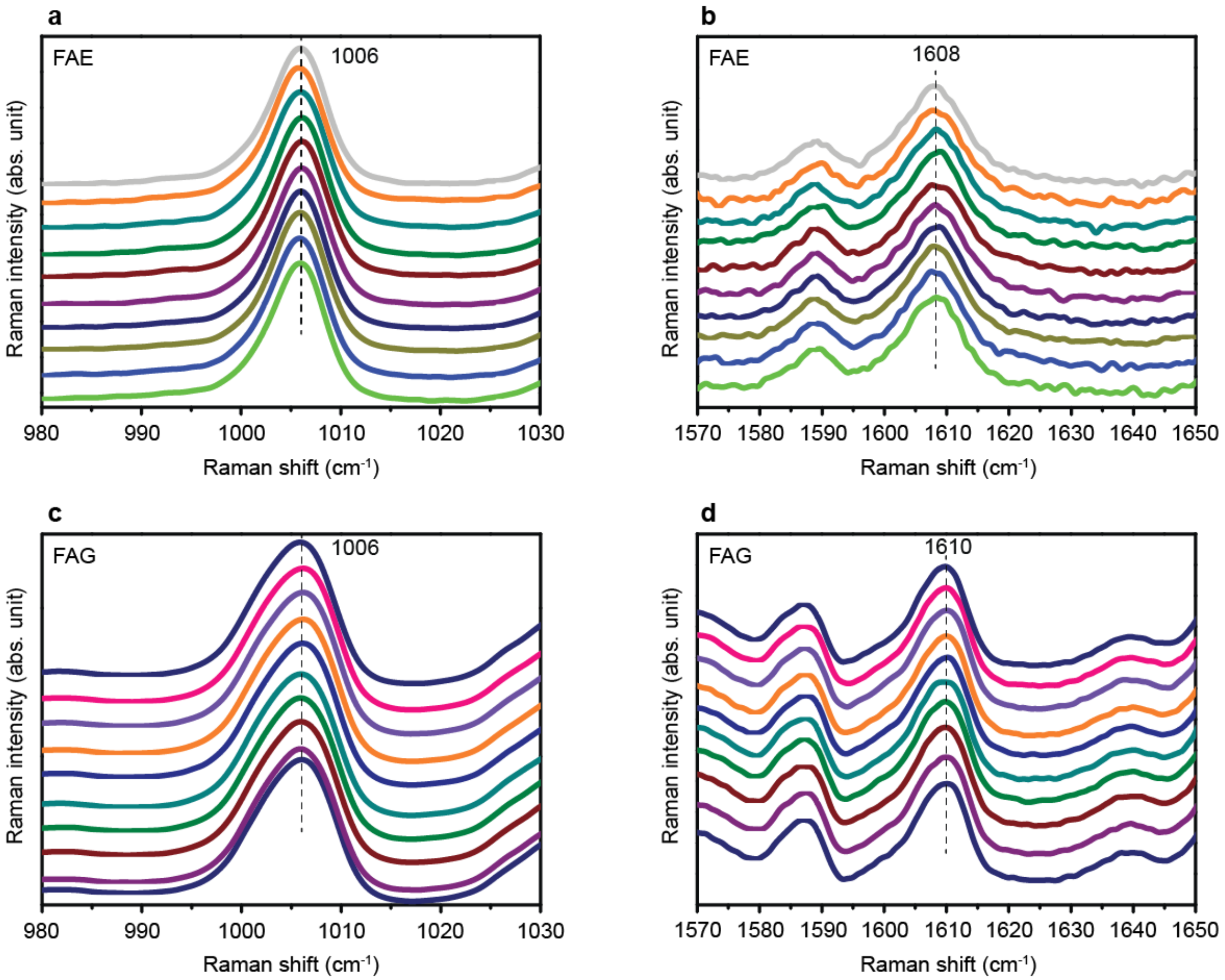

Supplementary Fig. 9. Ten superimposed Raman spectra of the truncated WT (FAE, top) and E22G mutant (FAG, bottom) tripeptides collected from ten different points of the same pair of tripeptide samples, supporting the high reproducibility of the wavenumber positions and shifts. In addition, the wavenumber positions and shifts match (within the experimental resolution) those shown in the Supplementary Fig. 8, collected from two different points of another pair of FAE/FAG samples.

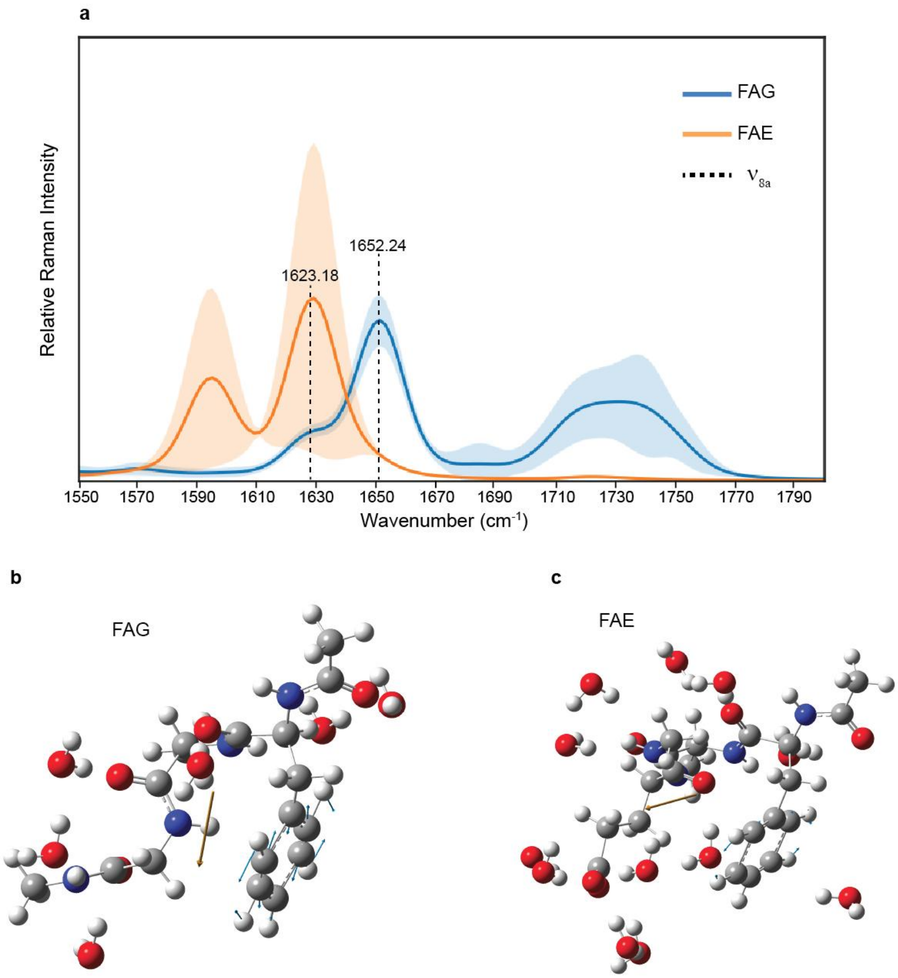

Supplementary Fig. 10. DFT-predicted Raman spectra of the truncated Aβ peptides are in qualitative agreement with the experimental data. a Computed Raman spectra of the truncated wild-type (WT) and E22G-Aβ peptides (FAE and FAG, respectively) show a blue shift from 1623 cm^{-1} (in FAE) to 1652 cm^{-1} (in FAG), following the same trend observed for the same mode of vibration (see Supplementary Fig. 8). The line graph presents multiple Raman spectra derived from two snapshots of the WT and nine snapshots of E22G tripeptides, extracted from MD trajectories, considering NH-π interactions within a geometrical distance d_{CN} and angle θ cutoff values (4 Å, 45°) along with interacting water molecules in the first hydration shell (4 Å). The Gaussian convoluted spectra are displayed, the relative intensity is scaled for an easier comparison of the observed blue shift, with shaded regions representing spectral variability across different snapshots for both peptides. **b,c** Vibrational displacement vectors (in blue arrows) and dipole derivative unit vectors (in yellow arrows) calculated for the solvated FAG (**b**) and FAE (**c**) tripeptides, underlying the Raman band exhibiting a blue shift in (**a**). This vibrational mode corresponds to the in-plane ring stretching ν_{8a} vibration of the aromatic ring of phenylalanine detected in our Raman spectroscopy measurements. Note: In regard to the discrepancy between the experimentally observed (1–3 cm^{-1}, Supplementary Fig. 8) and theoretically predicted (~30 cm^{-1}) Raman shifts after convolution, it should be noted that the experimental measurements were performed on isolated peptide fragments, whereas the theoretical Raman spectra were derived from truncated

conformations selected from the simulated ensemble of full-length peptides. Furthermore, variations in the number, orientation, and positioning of water molecules in the first hydration shell included in the theoretical Raman spectra calculations may not precisely reflect experimental conditions, which could also contribute to the observed discrepancy. Nevertheless, we must highlight that our interpretation focuses on the consistency between experimental and theoretical results in reproducing the same qualitative blue-shift trend, rather than the absolute magnitudes of the shifts.

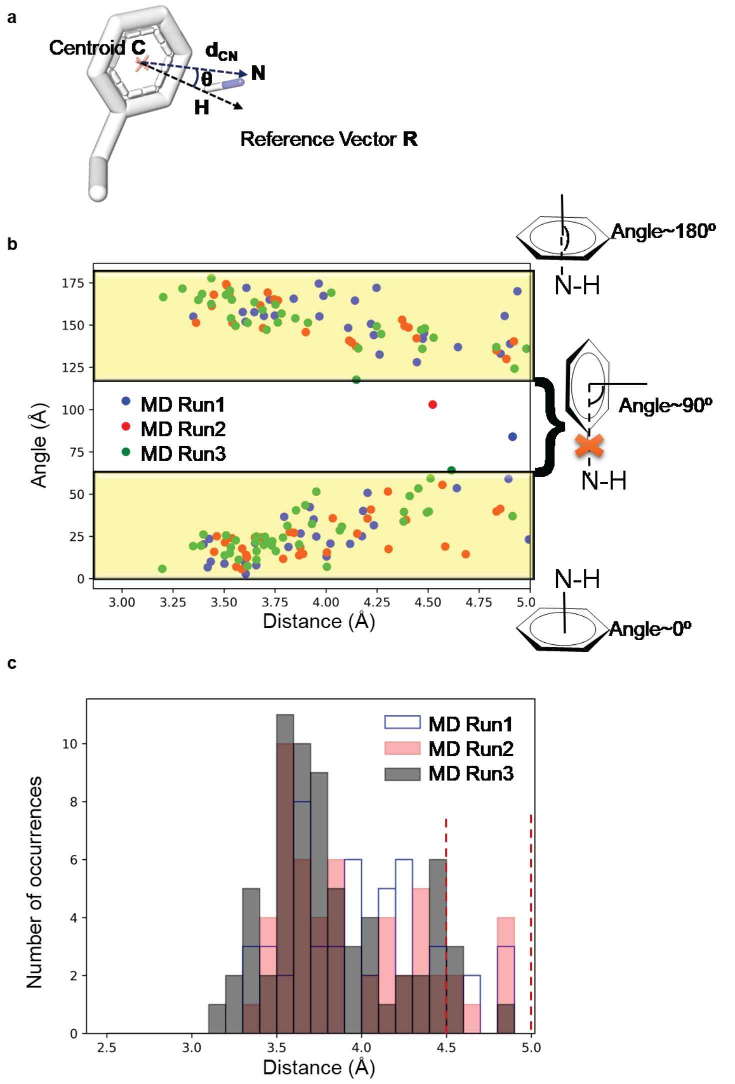

Supplementary Fig. 11. Statistical analysis performed for the selection of geometric cutoff criteria for NH-π interaction and their representative visualization. a Three-dimensional visualization depicting the geometric relationship among vectors relative to aryl residues and the N-H bond. The reference vector R, normal to the ring and emanating from the centroid C, is illustrated as a black dashed arrow, while the vector connecting N and the aromatic ring centroid is depicted in blue, serving as the basis for geometric computations. **b** Displayed is a scatter plot showcasing distances and angles between Phe20 and Gly22 across all frames captured from three replicates of MD simulations for the E22G-Aβ40. Regions of most interactions are highlighted in yellow, with cartoon representations at extremes (0°, 90°, and 180°) by the side of the plot. **c** Distribution plot illustrating occurrences against distances ranging up to 5 Å between the centroid and N, with a bin size of 0.1 Å. This plot encompasses data from three independent MD simulation runs of the E22G-Aβ40, specifically focusing on angles within the 0°-60° (θ, between the CN and R vectors) and 120°-180° (ϕ, between the CH and HN vectors) ranges. The two cutoff distances chosen for further analysis are demarcated by red dashed lines.

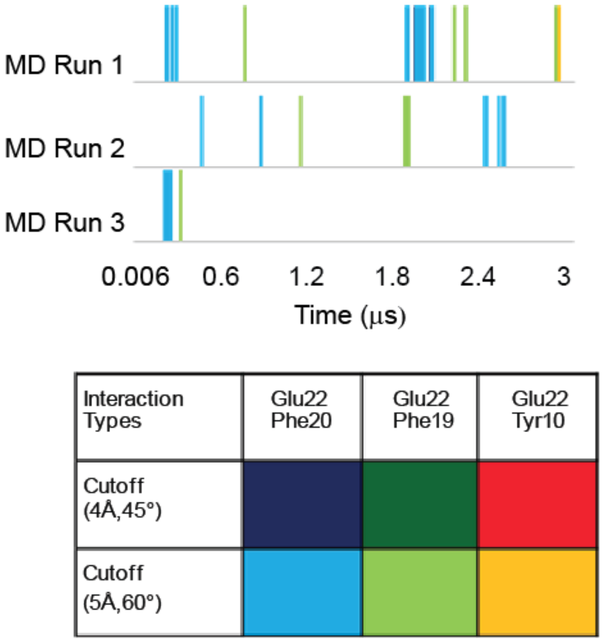

Supplementary Fig. 12. MD frames showcasing NH-π interactions observed in three independent MD runs of the wild-type Aβ40 (each 3 μs long). The same data for the MD runs of the E22G-Aβ40 peptide is shown in Fig. 3a. The color scheme represents interaction types and their respective geometric cutoff values for distance d_{CN} and angle θ (4 Å, 45º) and (5 Å, 60º) for a hydrogen bond interaction.

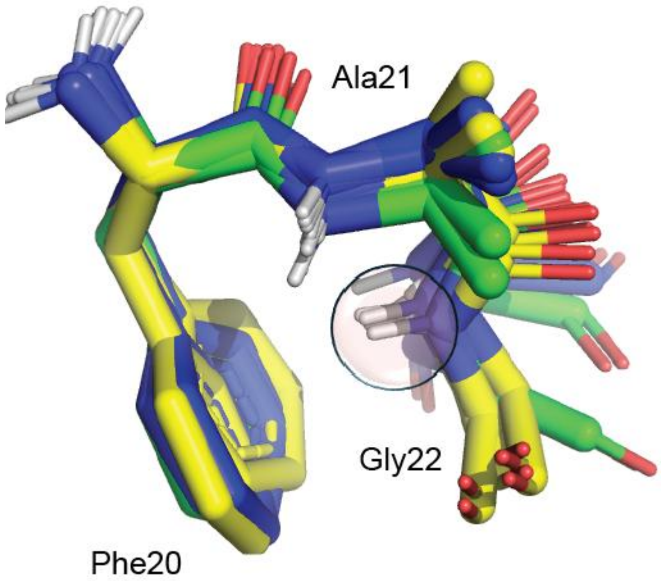

Supplementary Fig. 13. Superimposed structures of peptides extracted from multiple molecular dynamics (MD) simulations showing the Gly22-Phe20 interaction. Structures were obtained from three independent snapshots from each of the three replicates of MD simulations (Run 1 in blue, Run 2 in yellow and Run 3 in green) where the NH-π interaction is observed between Phe20 and Gly22 of E22G variant within the (4 Å, 45º) geometric cutoff. The average RMSD is 0.406 Å for the superimposed structures for E22G variant. The N-H group of interest is highlighted in black circle.

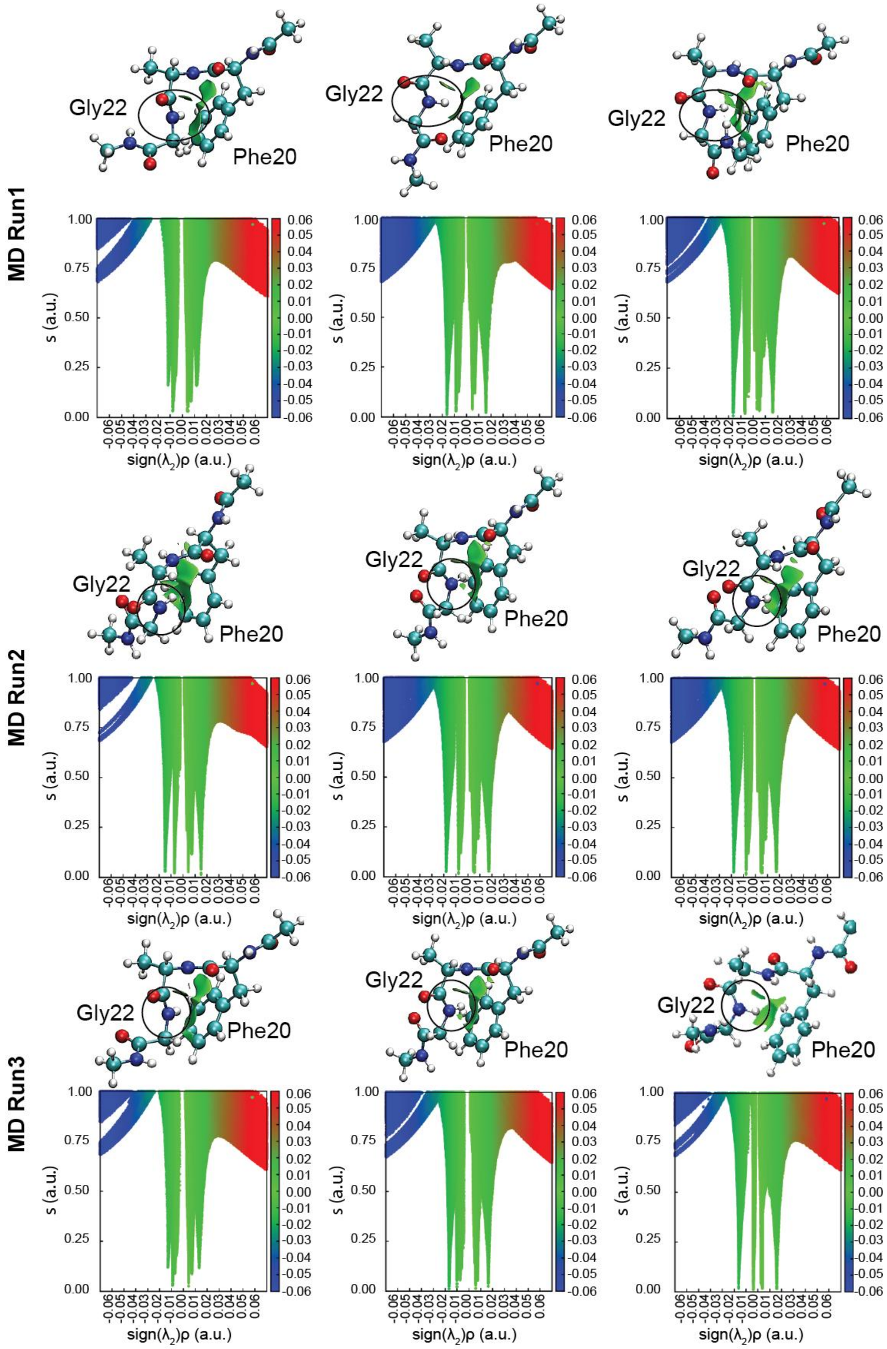

Supplementary Fig. 14. Non-covalent interaction (NCI) analysis of the truncated peptide structures showing the Gly22-Phe20 interaction in the E22G variant. NCI analysis performed on truncated capped peptide structures obtained from three independent snapshots from each of the three replicates of MD simulations where the NH-π interaction is observed between Phe20 and Gly22 of E22G variant within the (4 Å, 45º) cutoff shown here. Both the computed electron density distribution (ρ) of the different conformations of the truncated peptide structures and their respective reduced density gradient (RDG, *s*) versus $\mathrm{sign}(\lambda_2)\rho$ are shown (color scheme: blue, attractive interactions; green, weak interactions; red, repulsive interactions). The RDG isosurface is at *s* = 0.3 a.u.

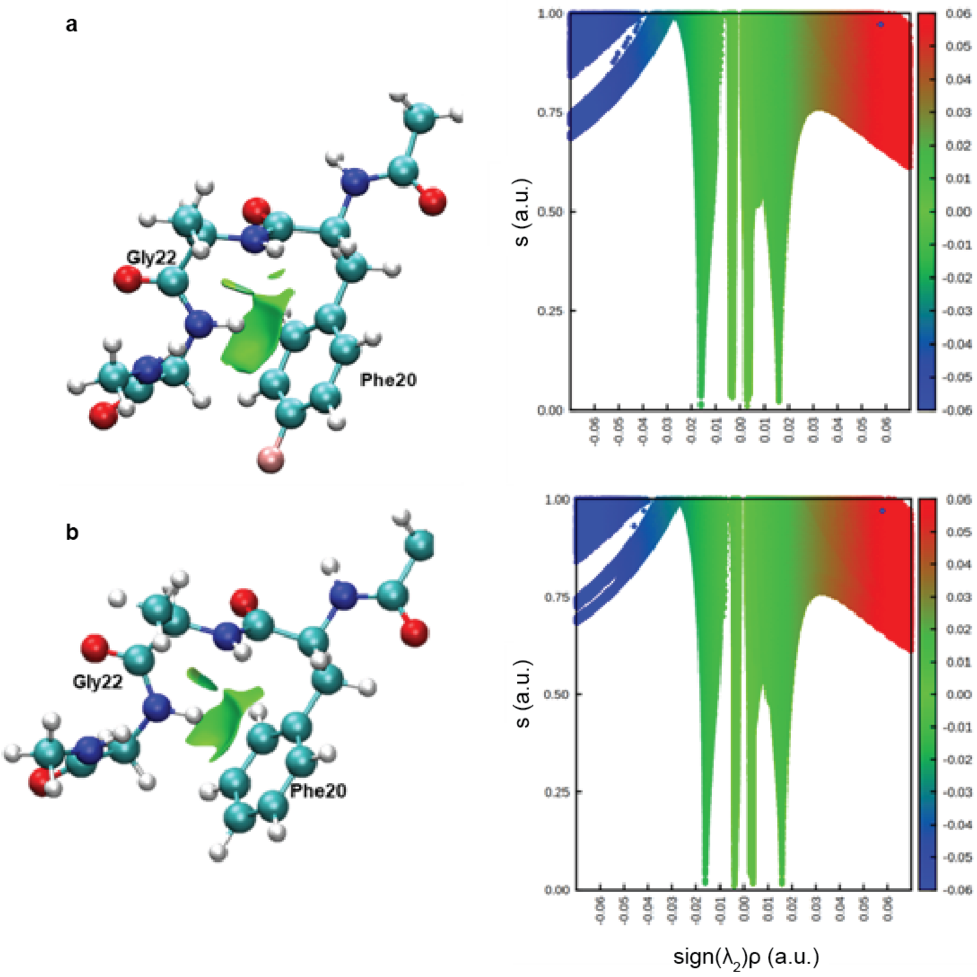

Supplementary Fig. 15. Fluorine substitution does not remove the NH-π interaction calculated for the E22G tripeptide model. a,b Non-Covalent Interactions (NCI) representation of electron density distribution (ρ) calculated for the mutated tripeptide (FAG), with (**a**) or without (**b**) fluorine substitution at the *para* position of Phe20. Right, the reduced density gradient (RDG, *s*) versus $\mathbf{sign}(\boldsymbol{\lambda_2})\boldsymbol{\rho}$ (see the text for definition; color scheme: blue, strong attractive interactions; green, weak interactions; red, strong repulsive interactions). Left, the RDG isosurfaces at $s = 0.3$ a.u.

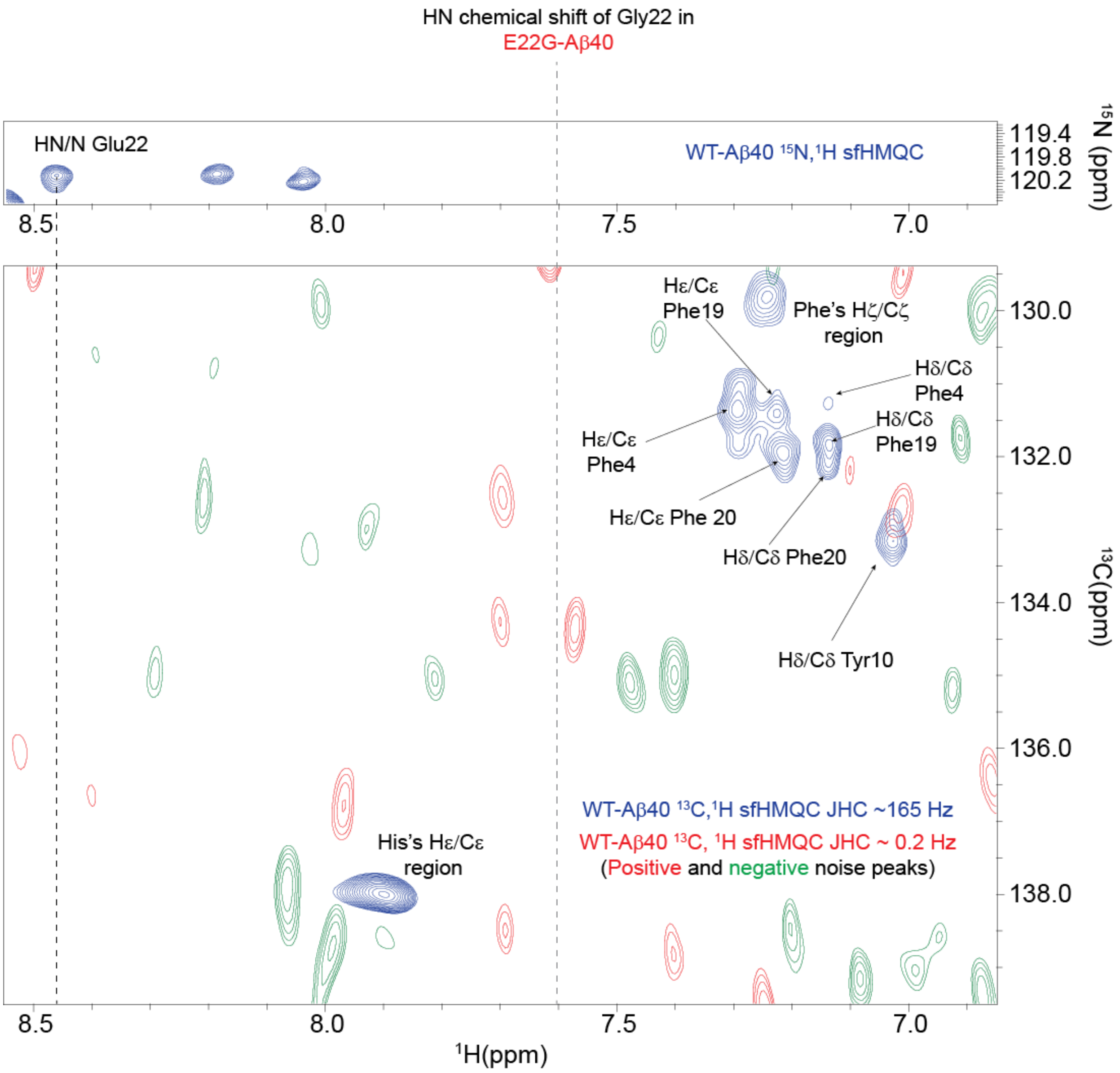

Supplementary Fig. 16. SOFAST-HMQC (sfHMQC) spectra of the control wild-type Aβ40 (WT-Aβ40) sample. Top panel: a region of the ^{15}N, ^{1}H sfHMQC spectrum of the WT-Aβ40 containing the HN/N cross-peak of Glu22. Bottom panel: control long-range ^{13}C, ^{1}H (based on $^{1}J_{HC}$ of ~ 0.2 Hz) sfHMQC spectrum of the WT-Aβ40 does not show any cross-peak between the HN of Glu22 (at ~ 8.46 ppm, marked by a dashed line) and aromatic carbons of Phe20, nor any cross-peak at the upfield chemical shift of the HN of Gly22 in the E22G-Aβ40 (at ~ 7.6 ppm, marked by another dashed line). The contour levels are adjusted to show the background noise (red: positive, green: negative). In the bottom panel, the short-range ^{13}C, ^{1}H (based on $^{1}J_{HC}$ of ~ 165 Hz) sfHMQC spectrum of the WT-Aβ40 containing the typical cross-peaks of directly attached ^{13}C and ^{1}H nuclei in aromatic side chains (in blue) is superimposed for comparison.

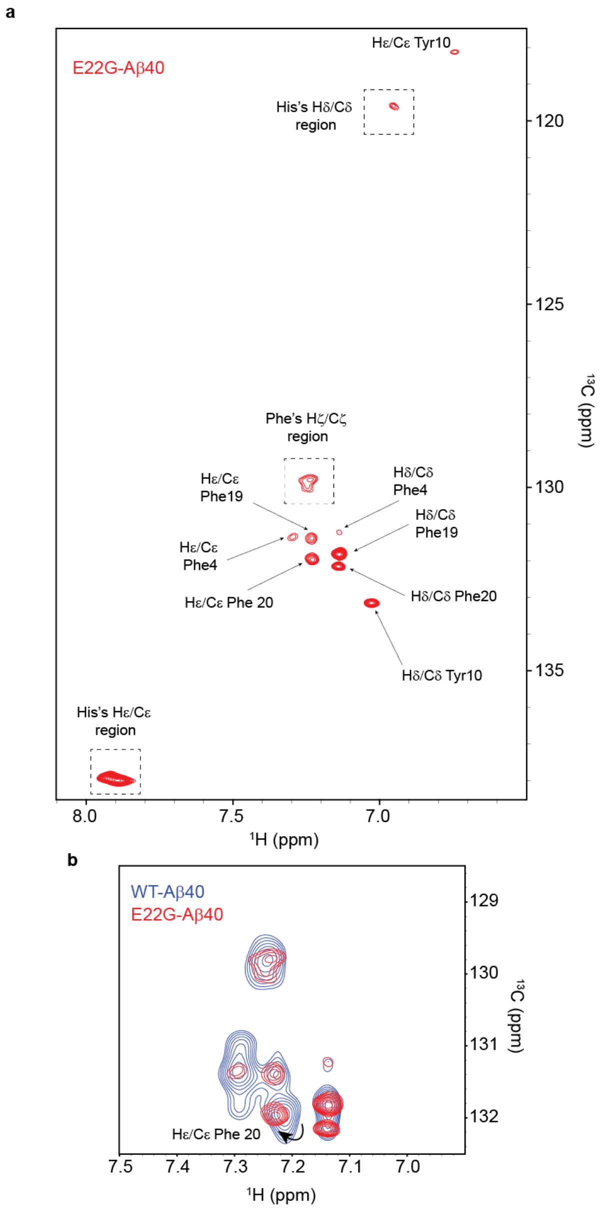

Supplementary Fig. 17. Assignment of ^{13}C aromatic chemical shifts assignment for the E22G-Aβ40. a Aromatic ^{13}C,^{1}H SOFAST-HMQC (sfHMQC) spectrum of the E22G-Aβ40 peptide, acquired using a short transfer delay of 3 ms (=1/2J, with $^{1}J_{HC}$ of 165 Hz). Assignments for three Phe and one Tyr rings are shown. The Hζ/Cζ cross-peaks of three Phe residues are not resolved and therefore could not be further assigned. The cross-peaks observed in the His region (Aβ has three His residues) are also shown. **b** Overlay of aromatic ^{13}C,^{1}H sfHMQC spectra of wild-type (WT) (blue) and E22G-Aβ40 (red). The arrow points to a small Hε chemical shift perturbation of Phe20, induced by E22G mutation.

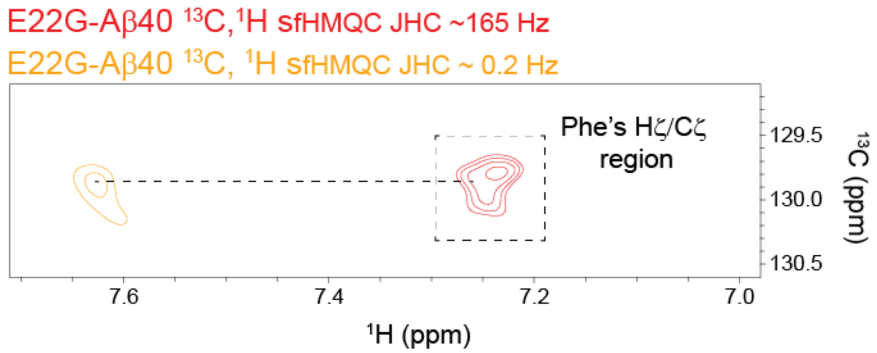

Supplementary Fig. 18. Tentative *J*-based correlation between the amide H of Gly22 and Cζ of Phe20's ring. A weak cross-peak (S:N ~ 2) between the amide H of Gly22 and Cζ of Phe20 (unresolved from other Phe residues) is observed in the long range $^{13}C,^{1}H$ SOFAST HMQC (sfHMQC) spectrum. The cross-peak of directly connected Hζ/Cζ nuclei, obtained through normal $^{13}C,^{1}H$ sfHMQC measurement, is shown for the comparison of Cζ chemical shift. Unlike other aromatic carbons (see Fig. 4), the poor signal-to-noise ratio does not allow an unequivocal assignment and distinction of the H^{N}-Cζ cross-peak from the noise.

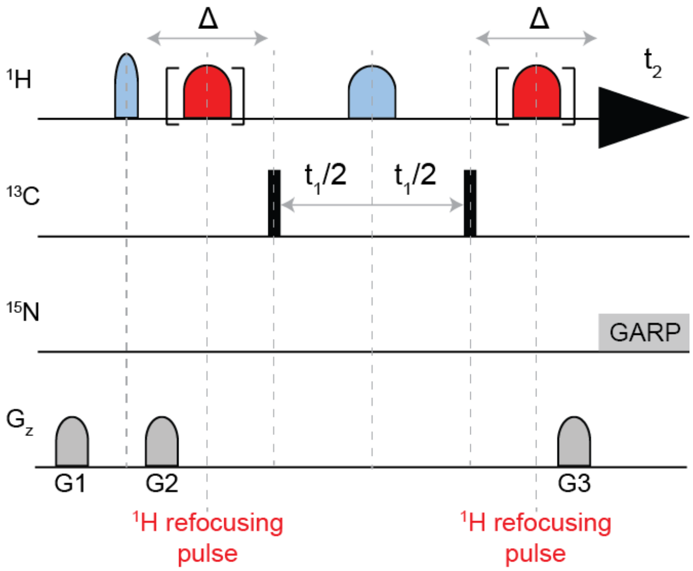

Supplementary Fig. 19. Modification of the ^{13}C, ^{1}H SOFAST-HMQC (sfHMQC) pulse sequence for the purpose of *J* coupling refocusing. The refocusing of *J* coupling is achieved through the introduction of two 180° proton pulses (in red) at the middle of the HMQC delays of the standard sfHMQC pulse sequence.

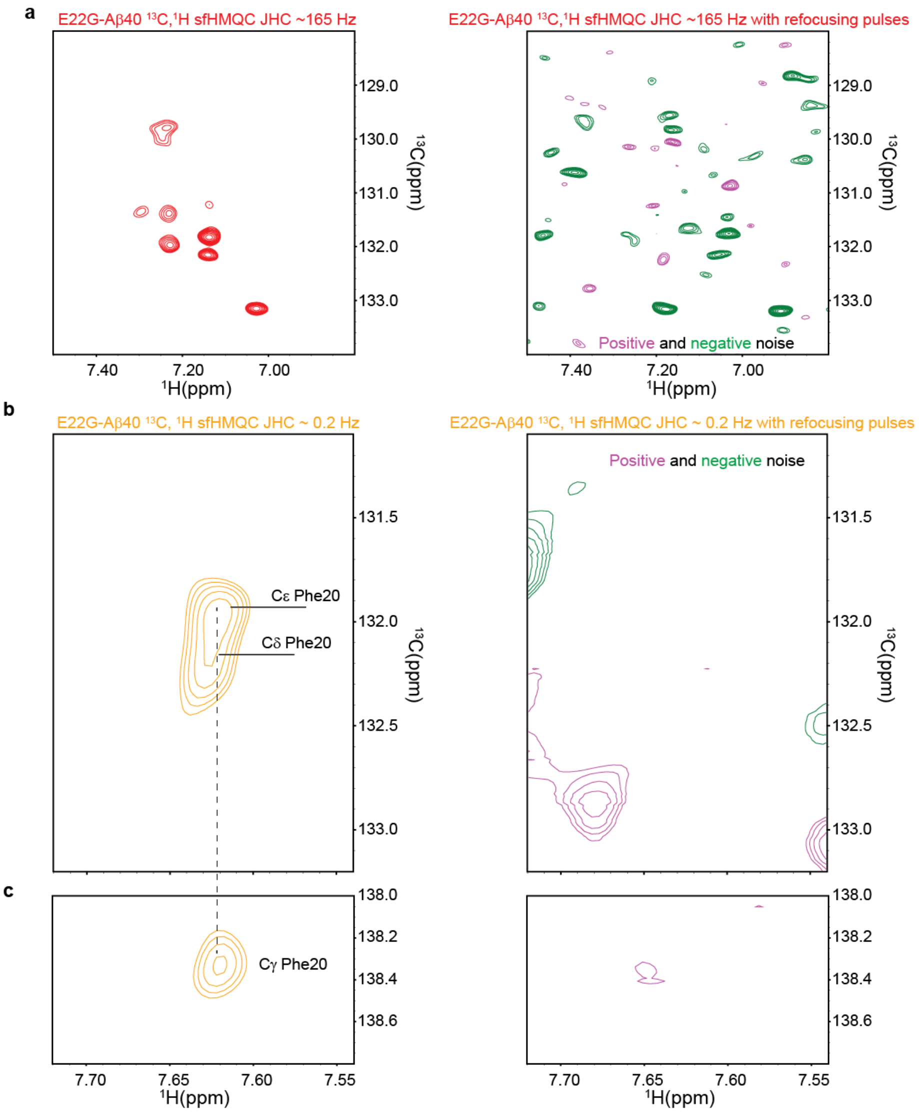

Supplementary Fig. 20. Refocusing of J coupling in the ^{13}C, ^{1}H SOFAST-HMQC (sfHMQC) experiments. a Short-range ^{13}C, ^{1}H (based on $^{1}J_{HC}$ of ~ 165 Hz) sfHMQC spectra of the E22G-Aβ40, obtained without (left) or with (right) J-refocusing pulses. As expected, introduction of refocusing pulses leads to the complete loss of the cross-peaks observed in the absence of them. **b,c** Long-range ^{13}C, ^{1}H (based on $^{1}J_{HC}$ of ~ 0.2 Hz) sfHMQC spectra of the E22G-Aβ40, obtained without (left) or with (right) refocusing pulses. The cross-peaks between the HN of Gly22 and Cδ/Cε (b) or Cγ (c) of Phe20 observed in the standard sfHMQC spectrum (left) are completely lost after introduction of J-refocusing pulses (right), supporting the role of J coupling as the mechanism underlying this correlation. The contour levels in the right spectra of panels a-c are adjusted to show the background noise (purple: positive, green: negative).

Supplementary Table 1. Optimized Cartesian coordinates (Å) for all the truncated E22G peptide (FAG) models studied here. The molecules were optimized without any symmetry constraint (C1 point group). Vibrational frequency analysis on the studied models confirms the minimum geometry on its corresponding potential surface by having zero imaginary frequency.

S1.1: Coordinates for the first truncated peptide model in Run 1 of MD simulation for E22G

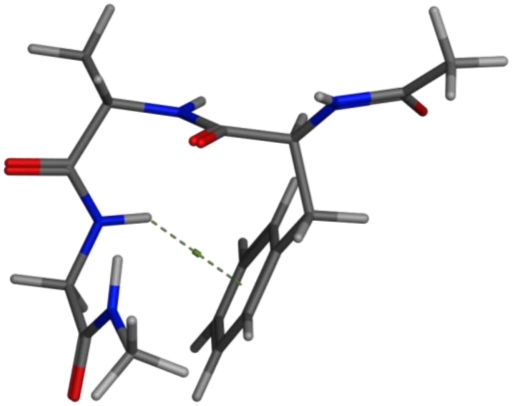

1 C1	5.8676	-0.1037	1.3341	C
2 C2	4.6442	-0.2384	0.4254	C
3 O3	4.6797	-0.9674	-0.5637	O
4 H4	6.6943	-0.7153	0.9726	H
5 H5	6.1719	0.9441	1.3550	H
6 H6	5.5916	-0.4206	2.3399	H
7 N7	3.4978	0.4149	0.7696	N
8 C8	2.2795	0.1489	0.0057	C
9 C9	1.7151	-1.2652	0.3106	C
10 C10	0.5660	-1.7006	-0.5758	C
11 C11	0.6062	-1.5369	-1.9715	C
12 C12	-0.4522	-1.9692	-2.7765	C
13 C13	-1.5646	-2.5934	-2.2022	C
14 C14	-1.6093	-2.7780	-0.8174	C
15 C15	-0.5576	-2.3260	-0.0148	C
16 C16	1.2198	1.2018	0.3323	C
17 O17	0.9015	1.4560	1.4994	O
18 H18	3.4122	0.8732	1.6683	H
19 H19	2.5546	0.2047	-1.0491	H
20 H20	1.4103	-1.2911	1.3612	H
21 H21	2.5477	-1.9647	0.1896	H
22 H22	1.4700	-1.0779	-2.4433	H
23 H23	-0.6093	-2.4590	1.0614	H
24 H24	-0.4035	-1.8226	-3.8515	H
25 H25	-2.4709	-3.2499	-0.3560	H
26 H26	-2.3871	-2.9292	-2.8264	H
27 N27	0.6091	1.7685	-0.7381	N
28 C28	-0.4367	2.7793	-0.6121	C
29 C29	-0.4900	3.6438	-1.8776	C
30 C30	-1.8315	2.1992	-0.3120	C
31 O31	-2.7229	2.9460	0.1052	O
32 H32	0.9593	1.5594	-1.6639	H
33 H33	-0.1981	3.4038	0.2534	H
34 H34	-1.2675	4.4026	-1.7712	H
35 H35	0.4706	4.1427	-2.0309	H
36 H36	-0.7179	3.0344	-2.7586	H
37 N37	-2.0115	0.8753	-0.5369	N

38 C38	-3.2627	0.1832	-0.2837	C
39 C39	-3.3128	-0.6096	1.0302	C
40 O40	-4.1929	-1.4665	1.1958	O
41 H41	-1.2309	0.3317	-0.8854	H
42 H42	-3.4589	-0.5235	-1.0914	H
43 H43	-4.0716	0.9181	-0.2631	H
44 N44	-2.3789	-0.3042	1.9471	N
45 C45	-2.2714	-0.9496	3.2381	C
46 H46	-1.7293	0.4350	1.7161	H
47 H47	-1.6948	-1.8704	3.1383	H
48 H48	-1.7692	-0.2826	3.9390	H
49 H49	-3.2679	-1.1849	3.6121	H

S1.2: Coordinates for the second truncated peptide model in Run 1 of MD simulation for E22G

1 C1	-6.3902	0.1052	-0.9540	C
2 C2	-5.0736	-0.3186	-0.3015	C
3 O3	-5.0544	-1.2169	0.5382	O
4 H4	-7.2254	-0.4815	-0.5712	H
5 H5	-6.5596	1.1609	-0.7424	H
6 H6	-6.3070	-0.0396	-2.0315	H
7 N7	-3.9503	0.3319	-0.6962	N
8 C8	-2.6447	-0.0032	-0.1454	C
9 C9	-2.1021	-1.3415	-0.7257	C
10 C10	-0.8804	-1.8667	-0.0052	C
11 C11	-0.9644	-2.2511	1.3445	C
12 C12	0.1547	-2.7412	2.0212	C
13 C13	1.3789	-2.8701	1.3539	C
14 C14	1.4729	-2.4987	0.0103	C
15 C15	0.3532	-1.9915	-0.6599	C
16 C16	-1.6875	1.1477	-0.4708	C
17 O17	-1.7571	1.7363	-1.5550	O
18 H18	-3.9793	1.0056	-1.4518	H
19 H19	-2.7588	-0.1134	0.9368	H
20 H20	-1.8953	-1.2010	-1.7912	H
21 H21	-2.9191	-2.0636	-0.6366	H
22 H22	-1.9145	-2.1725	1.8669	H
23 H23	0.4426	-1.6890	-1.6994	H
24 H24	0.0706	-3.0288	3.0651	H
25 H25	2.4165	-2.5852	-0.5183	H
26 H26	2.2492	-3.2553	1.8768	H
27 N27	-0.7528	1.4212	0.4750	N
28 C28	0.1616	2.5574	0.3766	C
29 C29	-0.2453	3.6722	1.3512	C
30 C30	1.6395	2.1763	0.5710	C
31 O31	2.4922	3.0695	0.6524	O
32 H32	-0.8735	1.0000	1.3882	H

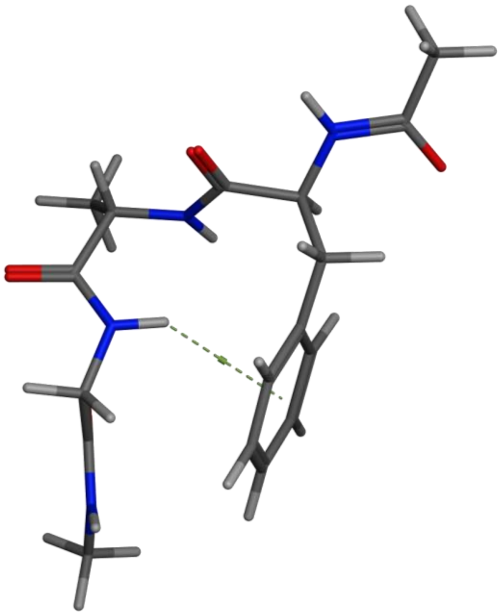

33 H33	0.0808	2.9263	-0.6499	H
34 H34	-1.2626	4.0018	1.1251	H
35 H35	-0.2144	3.3117	2.3850	H
36 H36	0.4362	4.5193	1.2578	H
37 N37	1.9337	0.8602	0.6570	N
38 C38	3.2881	0.3766	0.7933	C
39 C39	3.8999	-0.0746	-0.5433	C
40 O40	3.2381	-0.1374	-1.5853	O
41 H41	1.2116	0.1847	0.4389	H
42 H42	3.2939	-0.4776	1.4775	H
43 H43	3.8966	1.1691	1.2356	H
44 N44	5.1952	-0.4527	-0.4751	N
45 C45	5.8967	-0.9688	-1.6313	C
46 H46	5.7177	-0.2686	0.3703	H
47 H47	5.9324	-0.2041	-2.4077	H
48 H48	6.9132	-1.2448	-1.3478	H
49 H49	5.3752	-1.8474	-2.0117	H

S1.3: Coordinates for the third truncated peptide model in Run 1 of MD simulation for E22G

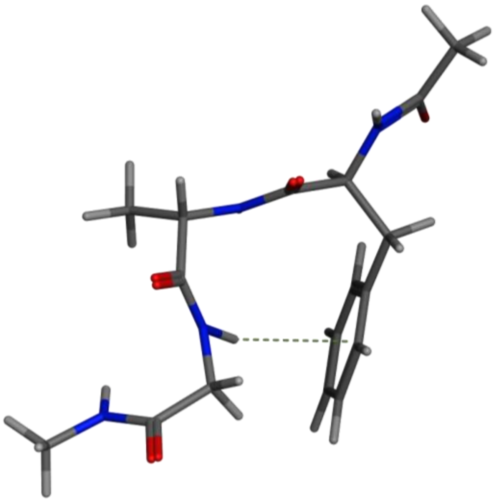

1 C1	6.2268	-1.5632	-0.7694	C
2 C2	5.0622	-0.7925	-0.1457	C
3 O3	5.2720	0.1939	0.5577	O
4 H4	7.1829	-1.1162	-0.4985	H
5 H5	6.1924	-2.5933	-0.4145	H
6 H6	6.1125	-1.5492	-1.8541	H
7 N7	3.7993	-1.2131	-0.4216	N
8 C8	2.6463	-0.4288	0.0124	C
9 C9	2.4784	0.8474	-0.8679	C
10 C10	1.5409	1.8999	-0.3132	C
11 C11	0.4135	2.3156	-1.0372	C
12 C12	-0.4216	3.3279	-0.5498	C
13 C13	-0.1478	3.9334	0.6798	C
14 C14	0.9728	3.5268	1.4126	C
15 C15	1.8108	2.5248	0.9169	C
16 C16	1.4090	-1.3268	-0.0787	C
17 O17	1.2393	-2.0722	-1.0501	O
18 H18	3.6327	-1.9545	-1.0914	H
19 H19	2.8246	-0.1251	1.0467	H
20 H20	2.1580	0.5398	-1.8681	H
21 H21	3.4797	1.2784	-0.9630	H
22 H22	0.1945	1.8536	-1.9965	H
23 H23	2.6902	2.2361	1.4870	H
24 H24	-1.2799	3.6470	-1.1344	H
25 H25	1.2004	3.9968	2.3651	H
26 H26	-0.7954	4.7179	1.0598	H
27 N27	0.5118	-1.2271	0.9344	N

28 C28	-0.6913	-2.0587	0.9869	C
29 C29	-1.1847	-2.1940	2.4333	C
30 C30	-1.8145	-1.5525	0.0595	C
31 O31	-2.5126	-2.3519	-0.5710	O
32 H32	0.7536	-0.6737	1.7465	H
33 H33	-0.4338	-3.0449	0.5936	H
34 H34	-2.0789	-2.8214	2.4623	H
35 H35	-0.4132	-2.6659	3.0482	H
36 H36	-1.4344	-1.2204	2.8666	H
37 N37	-2.0252	-0.2110	0.0173	N
38 C38	-2.9033	0.3818	-0.9815	C
39 C39	-4.4000	0.1020	-0.8311	C
40 O40	-5.1043	0.0614	-1.8518	O
41 H41	-1.3352	0.3926	0.4462	H
42 H42	-2.6369	0.0332	-1.9834	H
43 H43	-2.7564	1.4642	-0.9525	H
44 N44	-4.9061	-0.0560	0.4075	N
45 C45	-6.2996	-0.3938	0.6073	C
46 H46	-4.3088	0.0482	1.2160	H
47 H47	-6.6341	-1.0508	-0.1963	H
48 H48	-6.4170	-0.9032	1.5637	H
49 H49	-6.8993	0.5165	0.6048	H

S1.4: Coordinates for the first truncated peptide model in Run 2 of MD simulation for E22G

1 C1	-6.4216	-0.1870	-0.0763	C
2 C2	-4.9919	-0.5378	0.3401	C
3 O3	-4.7654	-1.5485	1.0029	O
4 H4	-7.1310	-0.9292	0.2893	H
5 H5	-6.6733	0.7908	0.3332	H
6 H6	-6.4673	-0.1467	-1.1656	H
7 N7	-4.0116	0.2914	-0.0945	N
8 C8	-2.6056	-0.0013	0.1429	C
9 C9	-2.1379	-1.2297	-0.6864	C
10 C10	-0.7576	-1.7548	-0.3515	C
11 C11	-0.3674	-2.0015	0.9764	C
12 C12	0.8914	-2.5343	1.2681	C
13 C13	1.7769	-2.8556	0.2345	C
14 C14	1.3947	-2.6312	-1.0909	C
15 C15	0.1451	-2.0727	-1.3786	C
16 C16	-1.8050	1.2408	-0.2537	C
17 O17	-2.0886	1.8758	-1.2752	O
18 H18	-4.2245	1.0313	-0.7526	H
19 H19	-2.4847	-0.2145	1.2079	H
20 H20	-2.1979	-0.9684	-1.7474	H
21 H21	-2.8722	-2.0196	-0.5018	H
22 H22	-1.0513	-1.7909	1.7938	H

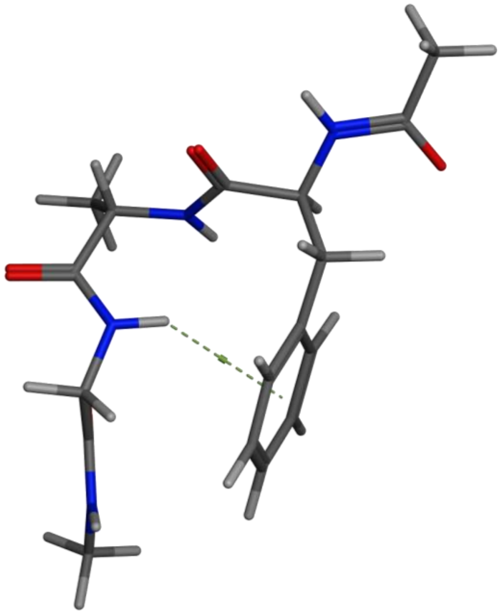

23 H23	-0.1355	-1.8874	-2.4120	H
24 H24	1.1761	-2.7051	2.3021	H
25 H25	2.0709	-2.8823	-1.9030	H
26 H26	2.7544	-3.2700	0.4596	H
27 N27	-0.7593	1.5492	0.5494	N
28 C28	0.1261	2.6841	0.3085	C
29 C29	0.5021	3.3517	1.6378	C
30 C30	1.4045	2.3140	-0.4668	C
31 O31	2.1979	3.2060	-0.7888	O
32 H32	-0.6483	1.0380	1.4160	H
33 H33	-0.4176	3.3928	-0.3218	H
34 H34	1.1583	4.2030	1.4497	H
35 H35	-0.4000	3.7011	2.1467	H
36 H36	1.0279	2.6479	2.2920	H
37 N37	1.5848	1.0060	-0.7574	N
38 C38	2.8534	0.4801	-1.2049	C
39 C39	3.7147	0.0448	-0.0091	C
40 O40	3.6076	0.5965	1.0932	O
41 H41	0.9124	0.3426	-0.3940	H
42 H42	3.3907	1.2714	-1.7351	H
43 H43	2.6858	-0.3457	-1.8991	H
44 N44	4.6122	-0.9313	-0.2485	N
45 C45	5.5820	-1.3196	0.7549	C
46 H46	4.6297	-1.3935	-1.1470	H
47 H47	6.3612	-1.9275	0.2924	H
48 H48	6.0308	-0.4276	1.1912	H
49 H49	5.0865	-1.8966	1.5359	H

S1.5: Coordinates for the second truncated peptide model in Run 2 of MD simulation for E22G

1 C1	6.3646	-0.2560	-0.5571	C
2 C2	4.8869	-0.5483	-0.8227	C
3 O3	4.5563	-1.4699	-1.5673	O
4 H4	7.0058	-0.9520	-1.0984	H
5 H5	6.5814	0.7629	-0.8772	H
6 H6	6.5510	-0.3481	0.5130	H
7 N7	3.9922	0.2326	-0.1658	N
8 C8	2.5665	-0.0634	-0.1650	C
9 C9	2.2622	-1.3187	0.7001	C
10 C10	0.8211	-1.7754	0.6707	C
11 C11	0.2174	-2.1732	-0.5345	C
12 C12	-1.1012	-2.6342	-0.5568	C
13 C13	-1.8356	-2.7217	0.6309	C
14 C14	-1.2479	-2.3253	1.8355	C
15 C15	0.0667	-1.8456	1.8516	C
16 C16	1.8429	1.1581	0.4070	C
17 O17	2.2920	1.7454	1.3973	O

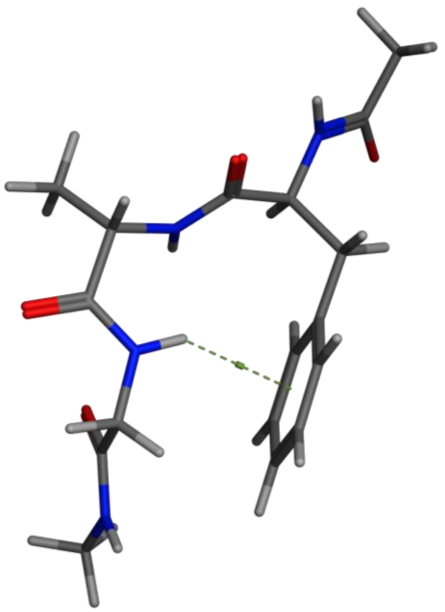

18 H18	4.3186	0.8806	0.5413	H
19 H19	2.2584	-0.2516	-1.1967	H
20 H20	2.5740	-1.1043	1.7268	H
21 H21	2.9067	-2.1162	0.3175	H
22 H22	0.7846	-2.1333	-1.4608	H
23 H23	0.5146	-1.5335	2.7911	H
24 H24	-1.5543	-2.9289	-1.4986	H
25 H25	-1.8118	-2.3812	2.7622	H
26 H26	-2.8586	-3.0835	0.6131	H
27 N27	0.6805	1.4918	-0.2039	N
28 C28	-0.1159	2.6583	0.1709	C
29 C29	-0.0309	3.7498	-0.9023	C
30 C30	-1.5799	2.2919	0.4799	C
31 O31	-2.4507	3.1693	0.4889	O
32 H32	0.4678	1.0494	-1.0899	H
33 H33	0.3132	3.0324	1.1057	H
34 H34	-0.6197	4.6181	-0.6025	H
35 H35	1.0116	4.0501	-1.0355	H
36 H36	-0.4172	3.3832	-1.8591	H
37 N37	-1.8270	0.9966	0.7854	N
38 C38	-3.1699	0.4767	0.8876	C
39 C39	-3.6805	-0.0268	-0.4735	C
40 O40	-3.1880	0.3654	-1.5378	O
41 H41	-1.0725	0.3289	0.6876	H
42 H42	-3.8314	1.2813	1.2209	H
43 H43	-3.1980	-0.3208	1.6327	H
44 N44	-4.7133	-0.8930	-0.4247	N
45 C45	-5.3910	-1.3224	-1.6301	C
46 H46	-5.0957	-1.1602	0.4707	H
47 H47	-4.6699	-1.7738	-2.3132	H
48 H48	-6.1565	-2.0565	-1.3760	H
49 H49	-5.8580	-0.4635	-2.1121	H

S1.6: Coordinates for the third truncated peptide model in Run 2 of MD simulation for E22G

1 C1	6.3312	-0.0302	-1.1889	C
2 C2	4.8412	-0.3718	-1.1273	C
3 O3	4.3603	-1.2055	-1.8933	O
4 H4	6.8349	-0.6082	-1.9633	H
5 H5	6.4362	1.0338	-1.4026	H
6 H6	6.7799	-0.2513	-0.2196	H
7 N7	4.0687	0.2800	-0.2167	N
8 C8	2.6578	-0.0607	-0.0795	C
9 C9	2.4666	-1.3330	0.7955	C
10 C10	1.0387	-1.8282	0.8402	C
11 C11	0.4325	-2.3488	-0.3155	C
12 C12	-0.8892	-2.8009	-0.2898	C

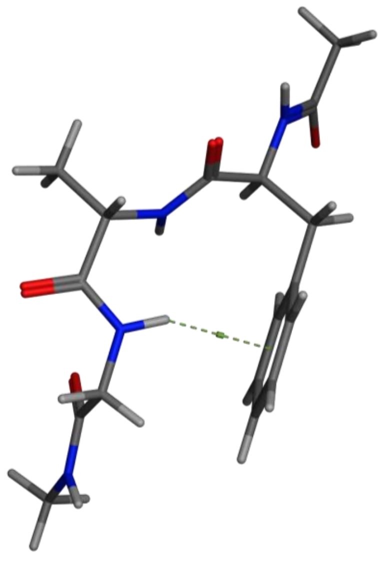

13 C13	-1.6251	-2.7515	0.8997	C
14 C14	-1.0329	-2.2355	2.0567	C
15 C15	0.2871	-1.7705	2.0229	C
16 C16	1.9061	1.1190	0.5382	C
17 O17	2.3843	1.7530	1.4846	O
18 H18	4.4778	0.9020	0.4693	H
19 H19	2.2787	-0.2703	-1.0825	H
20 H20	2.8329	-1.1161	1.8037	H
21 H21	3.1146	-2.1011	0.3618	H
22 H22	1.0009	-2.4037	-1.2405	H
23 H23	0.7376	-1.3621	2.9235	H
24 H24	-1.3431	-3.1946	-1.1944	H
25 H25	-1.5978	-2.1855	2.9829	H
26 H26	-2.6505	-3.1084	0.9216	H
27 N27	0.6712	1.3519	0.0219	N
28 C28	-0.1643	2.4739	0.4498	C
29 C29	0.0695	3.7104	-0.4262	C
30 C30	-1.6595	2.1018	0.5118	C
31 O31	-2.5206	2.9867	0.4485	O
32 H32	0.4442	0.9152	-0.8631	H
33 H33	0.1368	2.7008	1.4782	H
34 H34	1.1264	3.9858	-0.3865	H
35 H35	-0.2029	3.5040	-1.4661	H
36 H36	-0.5329	4.5475	-0.0695	H
37 N37	-1.9628	0.7962	0.6968	N
38 C38	-3.3278	0.3224	0.7295	C
39 C39	-3.8743	-0.0143	-0.6720	C
40 O40	-3.2502	0.2598	-1.7028	O
41 H41	-1.2163	0.1135	0.7179	H
42 H42	-3.9610	1.0987	1.1680	H
43 H43	-3.3816	-0.5600	1.3711	H
44 N44	-5.0962	-0.5955	-0.6896	N
45 C45	-5.7357	-0.9711	-1.9337	C
46 H46	-5.5131	-0.8923	0.1811	H
47 H47	-5.7165	-0.1251	-2.6226	H
48 H48	-5.2031	-1.8125	-2.3777	H
49 H49	-6.7697	-1.2568	-1.7398	H

S1.7: Coordinates for the first truncated peptide model in Run 3 of MD simulation for E22G

1 C1	6.2149	-1.4849	0.1831	C
2 C2	4.9780	-0.6860	0.5982	C
3 O3	5.0386	0.1208	1.5243	O
4 H4	7.0739	-1.2297	0.8028	H
5 H5	5.9937	-2.5476	0.2868	H
6 H6	6.4400	-1.2617	-0.8606	H
7 N7	3.8564	-0.9079	-0.1298	N

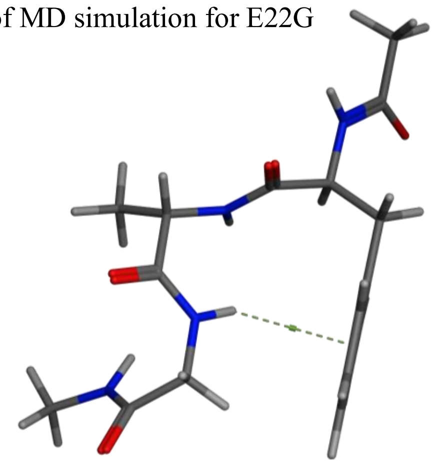

8	C8	2.6223	-0.1686	0.0997	C
9	C9	2.6972	1.2776	-0.4470	C
10	C10	1.4316	2.0677	-0.1979	C
11	C11	0.6246	2.4905	-1.2644	C
12	C12	-0.5372	3.2342	-1.0336	C
13	C13	-0.9194	3.5513	0.2734	C
14	C14	-0.1335	3.1181	1.3475	C
15	C15	1.0346	2.3878	1.1116	C
16	C16	1.5128	-0.9466	-0.6080	C
17	O17	1.5426	-1.1089	-1.8321	O
18	H18	3.9174	-1.4651	-0.9729	H
19	H19	2.4495	-0.1363	1.1788	H
20	H20	2.9098	1.2239	-1.5183	H
21	H21	3.5496	1.7569	0.0440	H
22	H22	0.9107	2.2394	-2.2821	H
23	H23	1.6485	2.0725	1.9515	H
24	H24	-1.1462	3.5567	-1.8730	H
25	H25	-0.4246	3.3562	2.3664	H
26	H26	-1.8227	4.1263	0.4550	H
27	N27	0.5247	-1.4310	0.1799	N
28	C28	-0.5699	-2.2302	-0.3551	C
29	C29	-1.0336	-3.2661	0.6781	C
30	C30	-1.7664	-1.3845	-0.8111	C
31	O31	-2.6494	-1.8909	-1.5110	O
32	H32	0.6063	-1.3289	1.1834	H
33	H33	-0.2041	-2.7393	-1.2501	H
34	H34	-1.8596	-3.8507	0.2687	H
35	H35	-0.2093	-3.9404	0.9241	H
36	H36	-1.3750	-2.7807	1.5988	H
37	N37	-1.8462	-0.1144	-0.3446	N
38	C38	-3.0137	0.7033	-0.6201	C
39	C39	-4.2991	0.1968	0.0423	C
40	O40	-5.3983	0.5075	-0.4387	O
41	H41	-1.0620	0.2863	0.1534	H
42	H42	-3.2138	0.7432	-1.6937	H
43	H43	-2.8119	1.7177	-0.2737	H
44	N44	-4.1532	-0.5669	1.1441	N
45	C45	-5.2576	-1.2834	1.7469	C
46	H46	-3.2157	-0.7583	1.4655	H
47	H47	-5.6108	-0.7380	2.6236	H
48	H48	-6.0702	-1.3737	1.0254	H
49	H49	-4.9270	-2.2773	2.0473	H

S1.8: Coordinates for the second truncated peptide model in Run 3 of MD simulation for E22G

1 C1	-6.3323	-0.2720	-0.9692	C
2 C2	-5.0693	-0.4844	-0.1331	C
3 O3	-4.9982	-1.4181	0.6635	O
4 H4	-7.0828	-1.0310	-0.7463	H
5 H5	-6.7363	0.7162	-0.7458	H
6 H6	-6.0647	-0.3243	-2.0245	H
7 N7	-4.0223	0.3463	-0.3758	N
8 C8	-2.6913	0.0468	0.1358	C
9 C9	-2.1055	-1.2091	-0.5760	C
10 C10	-0.7868	-1.7202	-0.0372	C
11 C11	0.2656	-2.0218	-0.9160	C
12 C12	1.4774	-2.5348	-0.4401	C
13 C13	1.6637	-2.7384	0.9293	C
14 C14	0.6217	-2.4462	1.8160	C
15 C15	-0.5941	-1.9521	1.3358	C
16 C16	-1.8035	1.2697	-0.1155	C
17 O17	-1.9733	1.9900	-1.1053	O
18 H18	-4.0707	1.0162	-1.1348	H
19 H19	-2.7764	-0.1485	1.2076	H
20 H20	-2.0131	-0.9807	-1.6425	H
21 H21	-2.8613	-1.9940	-0.4745	H
22 H22	0.1347	-1.8534	-1.9818	H
23 H23	-1.3988	-1.7546	2.0389	H
24 H24	2.2782	-2.7622	-1.1371	H
25 H25	0.7529	-2.6062	2.8821	H
26 H26	2.6103	-3.1178	1.3013	H
27 N27	-0.8045	1.4654	0.7787	N
28 C28	0.1361	2.5785	0.6895	C
29 C29	0.6042	2.9822	2.0937	C
30 C30	1.3597	2.2983	-0.2030	C
31 O31	2.1058	3.2342	-0.5170	O
32 H32	-0.7581	0.8669	1.5935	H
33 H33	-0.3851	3.4156	0.2175	H
34 H34	1.1184	2.1508	2.5877	H
35 H35	1.2969	3.8226	2.0227	H
36 H36	-0.2535	3.2798	2.7028	H
37 N37	1.5540	1.0197	-0.5867	N
38 C38	2.7199	0.5797	-1.3138	C
39 C39	3.7982	-0.0085	-0.3872	C
40 O40	3.8543	0.2829	0.8123	O
41 H41	0.9197	0.3104	-0.2431	H
42 H42	3.1527	1.4443	-1.8254	H
43 H43	2.4225	-0.1491	-2.0726	H
44 N44	4.6861	-0.8236	-0.9993	N
45 C45	5.8732	-1.3234	-0.3376	C

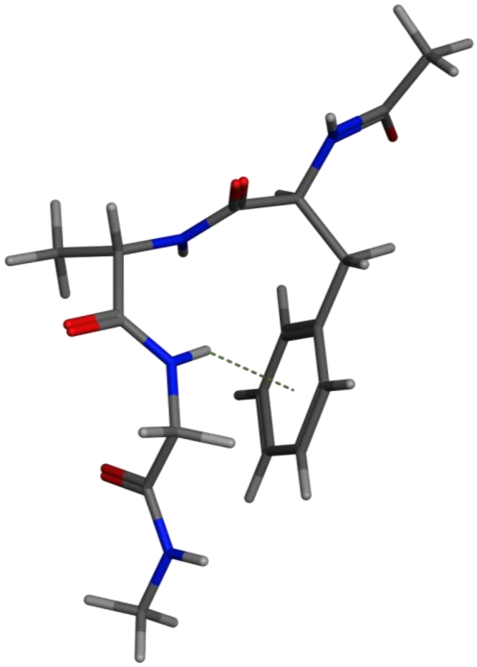

46 H46	4.5751	-0.9882	-1.9895	H
47 H47	6.1568	-0.6419	0.4666	H
48 H48	5.6703	-2.3100	0.0786	H
49 H49	6.6890	-1.3934	-1.0563	H

S1.9: Coordinates for the third truncated peptide model in Run 3 of MD simulation for E22G

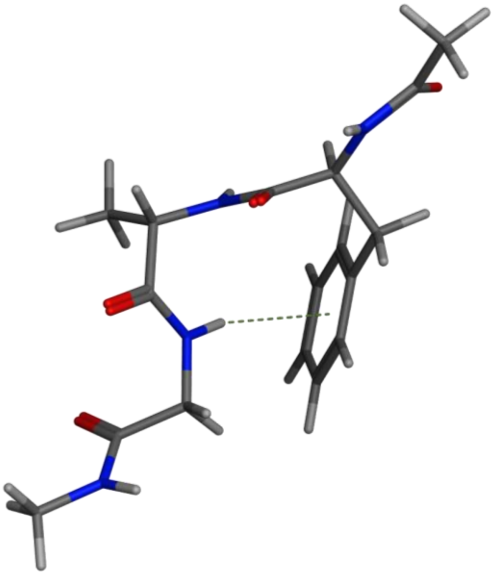

1 C1	5.9984	-1.9966	-1.0113	C
2 C2	5.0290	-0.9527	-0.4548	C
3 O3	5.4478	0.1172	-0.0157	O
4 H4	7.0289	-1.6470	-0.9499	H
5 H5	5.8908	-2.9151	-0.4331	H
6 H6	5.7408	-2.1914	-2.0526	H
7 N7	3.7120	-1.2695	-0.4807	N
8 C8	2.6904	-0.3587	0.0108	C
9 C9	2.4294	0.7993	-0.9946	C
10 C10	1.4698	1.8540	-0.4884	C
11 C11	0.3081	2.1728	-1.2070	C
12 C12	-0.5795	3.1501	-0.7412	C
13 C13	-0.3256	3.8134	0.4628	C
14 C14	0.8328	3.5077	1.1858	C
15 C15	1.7248	2.5438	0.7091	C
16 C16	1.4183	-1.1775	0.2421	C
17 O17	1.1247	-2.1238	-0.4984	O
18 H18	3.3940	-2.1260	-0.9181	H
19 H19	3.0458	0.0649	0.9541	H
20 H20	2.0641	0.3667	-1.9311	H
21 H21	3.4063	1.2499	-1.1968	H
22 H22	0.0958	1.6524	-2.1372	H
23 H23	2.6291	2.3315	1.2735	H
24 H24	-1.4671	3.3924	-1.3185	H
25 H25	1.0450	4.0234	2.1177	H
26 H26	-1.0183	4.5643	0.8302	H
27 N27	0.6263	-0.7865	1.2666	N
28 C28	-0.6001	-1.5097	1.5964	C
29 C29	-1.1166	-1.0692	2.9706	C
30 C30	-1.6953	-1.3321	0.5314	C
31 O31	-2.4522	-2.2666	0.2433	O
32 H32	0.9304	-0.0337	1.8707	H
33 H33	-0.3783	-2.5801	1.6048	H
34 H34	-1.3403	0.0024	2.9820	H
35 H35	-2.0315	-1.6143	3.2137	H
36 H36	-0.3697	-1.2843	3.7401	H
37 N37	-1.8160	-0.0996	-0.0044	N
38 C38	-2.8288	0.2088	-0.9920	C

39 C39	-4.2479	-0.0734	-0.4694	C
40 O40	-4.5668	0.1653	0.7002	O
41 H41	-1.1407	0.6151	0.2318	H
42 H42	-2.6396	-0.3495	-1.9159	H
43 H43	-2.7569	1.2737	-1.2233	H
44 N44	-5.1002	-0.5500	-1.4054	N
45 C45	-6.4287	-1.0362	-1.0937	C
46 H46	-4.6946	-0.7809	-2.3015	H
47 H47	-7.1170	-0.7502	-1.8904	H
48 H48	-6.4079	-2.1220	-1.0060	H
49 H49	-6.7635	-0.6014	-0.1519	H

Supplementary Table 2. Density Functional Theory (DFT) analysis of indirect spin-spin scalar (*J*) coupling between the amide H of Gly22 and aromatic carbons of Phe20. Individual contributions of the Fermi contact (FC), spin-dipolar (SD) and paramagnetic and diamagnetic spin-orbit (PSO, DSO) mechanisms and total *J* values are reported at five different levels of theory (density functional/basis set). The predicted total *J* couplings lie within the range of ~ 0.10 to 0.26 Hz. The chemical structure was drawn using the software ChemDraw (RRID: SCR_016768).

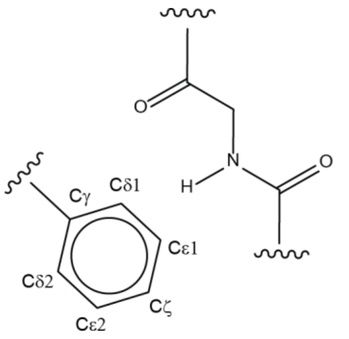

	**H-**Cγ	**H-**Cδ1	**H-**Cε1	**H-**Cζ	**H-**Cε2	**H-**Cδ2
**FC (mHz)**						
B3LYP/6-31++g**	2.601	-12.817	3.414	2.262	7.449	16.436
B3LYP/6-311++g**	-5.687	-3.693	-1.250	-1.374	-2.006	-10.112
PBE1PBE/6-311++g**	-3.761	-6.083	-2.194	-1.477	-0.621	-6.781
PBE1PBE/cc-pVTZ	5.755	2.839	1.212	0.435	2.197	1.024
PBE1PBE/aug-cc-pVTZ	-5.774	-0.980	1.224	-0.514	1.419	-3.654
wB97XD/aug-cc-pVTZ	2.549	28.124	18.315	5.617	-18.146	-31.680
**SD (mHz)**						
B3LYP/6-31++g**	0.574	-10.155	9.344	14.479	16.850	11.300
B3LYP/6-311++g**	-0.022	-10.002	10.551	15.515	18.386	12.690
PBE1PBE/6-311++g**	-0.133	-10.555	10.803	15.381	18.598	12.352
PBE1PBE/cc-pVTZ	1.233	-8.888	11.931	16.138	19.633	13.238
PBE1PBE/aug-cc-pVTZ	0.651	-10.203	11.014	15.722	18.718	12.719
wB97XD/aug-cc-pVTZ	9.076	6.699	14.830	13.798	10.397	8.019
**PSO (mHz)**						
B3LYP/6-31++g**	-340.448	-291.486	-219.681	-210.315	-240.26	-289.936
B3LYP/6-311++g**	-421.069	-373.391	-288.566	-272.147	-306.554	-363.652
PBE1PBE/6-311++g**	-421.901	-373.287	-288.316	-271.792	-306.307	-363.696
PBE1PBE/cc-pVTZ	-437.972	-387.346	-298.15	-279.946	-318.102	-377.106
PBE1PBE/aug-cc-pVTZ	-446.472	-391.465	-298.585	-279.826	-317.018	-380.676
wB97XD/aug-cc-pVTZ	-564.952	-450.891	-369.323	-396.409	-490.665	-565.922
**DSO (mHz)**						
B3LYP/6-31++g**	593.66	530.878	407.922	360.961	397.375	488.026
B3LYP/6-311++g**	592.493	530.039	407.492	360.392	396.447	486.735
PBE1PBE/6-311++g**	592.949	530.278	407.558	360.511	396.728	487.202
PBE1PBE/cc-pVTZ	595.558	532.248	408.701	361.582	398.342	489.491
PBE1PBE/aug-cc-pVTZ	595.647	532.372	408.761	361.658	398.369	489.534
wB97XD/aug-cc-pVTZ	745.988	591.515	492.459	528.897	656.207	748.521
**Total J (mHz)**						
B3LYP/6-31++g**	256.388	216.421	200.999	167.387	181.415	211.033
B3LYP/6-311++g**	165.715	142.953	128.226	102.386	106.273	125.661
PBE1PBE/6-311++g**	167.154	140.354	127.851	102.623	108.398	129.078
PBE1PBE/cc-pVTZ	164.574	138.852	123.693	98.209	102.071	126.647
PBE1PBE/aug-cc-pVTZ	144.052	129.724	122.414	97.040	101.488	117.923
wB97XD/aug-cc-pVTZ	192.660	175.447	156.282	151.903	157.793	158.938

Supplementary Table 3. Density Functional Theory (DFT) analysis of indirect spin-spin scalar (*J*) coupling between the amide N of Gly22 and aromatic carbons of Phe20. Individual contributions of the Fermi contact (FC), spin-dipolar (SD) and paramagnetic and diamagnetic spin-orbit (PSO, DSO) mechanisms and total *J* values are reported at five different levels of theory (density functional/basis set). The predicted total *J* couplings lie within the range of ~ −0.01 to 0.02 Hz. The chemical structures was drawn using the software ChemDraw (RRID: SCR_016768).

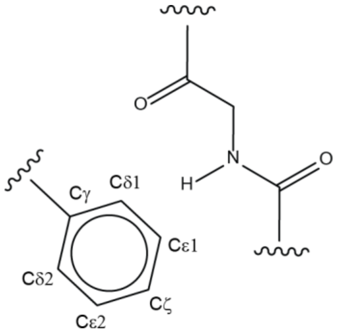

	**N-**Cγ	**N-**Cδ1	**N-**Cε1	**N-**Cζ	**N-**Cε2	**N-**Cδ2
**FC (mHz)**						
B3LYP/6-31++g**	12.203	16.263	4.276	-2.716	1.039	-1.219
B3LYP/6-311++g**	8.322	19.622	-0.253	-6.134	-0.610	-2.475
PBE1PBE/6-311++g**	9.120	18.015	0.021	-5.543	-0.567	-2.534
PBE1PBE/cc-pVTZ	0.912	7.076	-3.594	-3.577	-0.456	-2.521
PBE1PBE/aug-cc-pVTZ	5.780	13.042	-1.549	-4.316	-0.812	-2.710
wB97XD/aug-cc-pVTZ	5.914	11.994	-11.321	-20.539	13.524	10.303
**SD (mHz)**						
B3LYP/6-31++g**	3.492	-0.628	-0.176	-0.037	1.434	1.844
B3LYP/6-311++g**	3.722	-0.958	-0.330	-0.100	1.523	2.063
PBE1PBE/6-311++g**	3.411	-1.010	-0.465	-0.104	1.524	2.006
PBE1PBE/cc-pVTZ	3.484	-1.002	-0.416	-0.099	1.549	2.063
PBE1PBE/aug-cc-pVTZ	3.545	-1.022	-0.487	-0.086	1.556	2.071
wB97XD/aug-cc-pVTZ	5.585	0.047	-1.087	-0.027	1.148	4.638
**PSO (mHz)**						
B3LYP/6-31++g**	-12.526	-14.524	-8.738	-6.352	-7.549	-9.762
B3LYP/6-311++g**	-14.574	-16.863	-10.579	-7.729	-9.005	-11.461
PBE1PBE/6-311++g**	-14.551	-16.664	-10.585	-7.722	-8.951	-11.378
PBE1PBE/cc-pVTZ	-14.553	-17.188	-11.034	-7.783	-8.848	-11.185
PBE1PBE/aug-cc-pVTZ	-15.349	-17.787	-11.490	-8.266	-9.315	-11.816
wB97XD/aug-cc-pVTZ	-20.907	-21.002	-14.046	-11.146	-16.371	-19.421
**DSO (mHz)**						
B3LYP/6-31++g**	18.787	22.287	19.169	13.799	11.912	13.208
B3LYP/6-311++g**	18.813	22.291	19.165	13.804	11.927	13.228
PBE1PBE/6-311++g**	25.242	22.283	19.156	13.797	11.919	13.223
PBE1PBE/cc-pVTZ	18.842	22.341	19.200	13.815	11.933	13.241
PBE1PBE/aug-cc-pVTZ	18.852	22.358	19.216	13.829	11.941	13.247
wB97XD/aug-cc-pVTZ	25.531	25.103	23.862	21.190	20.310	20.280
**Total J (mHz)**						
B3LYP/6-31++g**	21.956	23.398	14.531	4.694	6.837	4.071
B3LYP/6-311++g**	16.283	24.093	8.003	-0.160	3.834	1.354
PBE1PBE/6-311++g**	23.222	22.624	8.127	0.428	3.927	1.316
PBE1PBE/cc-pVTZ	8.685	11.227	4.157	2.356	4.179	1.598
PBE1PBE/aug-cc-pVTZ	12.828	16.590	5.690	1.160	3.370	0.791
wB97XD/aug-cc-pVTZ	14.245	16.142	-2.591	-10.522	18.611	15.799

Supplementary Table 4. Publicly available biological databases of Intrinsically Disordered Proteins (IDPs) or Intrinsically Disordered Regions (IDRs) used to extract the sequences of IDPs/IDRs for searching motif identified from Aβ40. Only the public databases with distinct IDP/IDR labelling mentioned in the review by Piovesan *et al.* [1] were used for our analyses.

Name of Database	URL	IDPs Retrieved	Date of Retrieval	Reference
DisProt	https://disprot.org/	11667	17 July, 2024	[2]
IDEAL	https://www.ideal-db.org/	1110	21 Nov., 2023	[3]
FuzDB	https://fuzdb.org/	404	16 Nov., 2023	[4]
MFIB	https://mfib.enzim.ttk.mta.hu/	529	21 Nov., 2023	[5]
DIBS	https://dibs.enzim.ttk.mta.hu/	529	21 Nov., 2023	[6]

Supplementary Table 5. The general Molecular Dynamics (MD) simulation setup for the wild-type (WT) and E22G Aβ40. Further details are provided in the Methods section.

	WT (no. atoms)	E22G (no. atoms)
Protein	598	590
Ion (sodium)	3	2
Water	70424	70472
Total	71025	71064
Simulation box dimensions	$15 \times 6 \times 6\ (nm)^3$	$15 \times 6 \times 6\ (nm)^3$

Supplementary Note

Nanosecond dynamics of aromatic rings in Aβ40: ^{19}F T_1 relaxation measurements

Assuming a reduced ^{19}F chemical shift anisotropy of 51-60 ppm for fluorine-labeled phenylalanine [7,8], the T_1 values reported in the Supplementary Fig. 6 correspond to rotational correlation time (τ_c) of 0.64-1.2 ns for Phe4 and Phe20 and 0.8-1.8 ns for Phe19. These τ_c values lie on the right side of the T_1 minimum point at the ^{19}F Larmor frequency of 565 MHz used here, hence an increase (decrease) in τ_c leads to a rise (drop) in T_1. Upon E22G mutation, the ^{19}F T_1 of Phe4 and Tyr10 far from the mutation site remained unaffected. Interestingly, however, the ^{19}F T_1 of Phe19 decreased from 0.74±0.01 s to 0.68±0.04 s, whereas that of Phe20 increased from 0.68±0.02 s to 0.73±0.02 s (Supplementary Fig. 6). The observed changes in ^{19}F T_1 of Phe19 and Phe20 are small but marginally significant and suggest slight mobilization of Phe19 along with rigidification of Phe20 through the E22G mutation.

References

1. Piovesan, D., Monzon, A. M., Quaglia, F. & Tosatto, S. C. Databases for intrinsically disordered proteins. *Acta Crystallogr. D* **78**, 144-151 (2022). https://doi.org/10.1107/S2059798321012109

2. Quaglia F. et al. DisProt in 2022: improved quality and accessibility of protein intrinsic disorder annotation. *Nucleic Acids Res.* **50**, D480-D487 (2022). https://doi.org/10.1093/nar/gkab1082

3. Fukuchi S. et al. IDEAL in 2014 illustrates interaction networks composed of intrinsically disordered proteins and their binding partners. *Nucleic Acids Res.* **42**, D320-D325 (2014). https://doi.org/10.1093/nar/gkt1010

4. Miskei, M., Antal, C. & Fuxreiter, M. FuzDB: database of fuzzy complexes, a tool to develop stochastic structure-function relationships for protein complexes and higher-order assemblies. *Nucleic Acids Res.* **45**, D228-D235 (2016). https://doi.org/10.1093/nar/gkw1019

5. Fichó, E., Reményi, I., Simon, I. & Mészáros, B. MFIB: a repository of protein complexes with mutual folding induced by binding. *Bioinformatics* **33**, 3682-3684 (2017). https://doi.org/10.1093/bioinformatics/btx486

6. Schad, E. et al. DIBS: a repository of disordered binding sites mediating interactions with ordered proteins. *Bioinformatics* **34**, 535-537 (2018). https://doi.org/10.1093/bioinformatics/btx640

7. Hull, W. E. & Sykes, B. D. Fluorotyrosine alkaline phosphatase: internal mobility of individual tyrosines and the role of chemical shift anisotropy as a 19F nuclear spin relaxation mechanism in proteins. *J. Mol. Biol.* **98**, 121-153 (1975). https://doi.org/10.1016/s0022-2836(75)80105-7

8. Durr, U. H., Grage, S. L., Witter, R. & Ulrich, A. S. Solid state 19F NMR parameters of fluorine-labeled amino acids. Part I: aromatic substituents. *J. Magn. Reson.* **191**, 7-15 (2008). https://doi.org/10.1016/j.jmr.2007.11.017

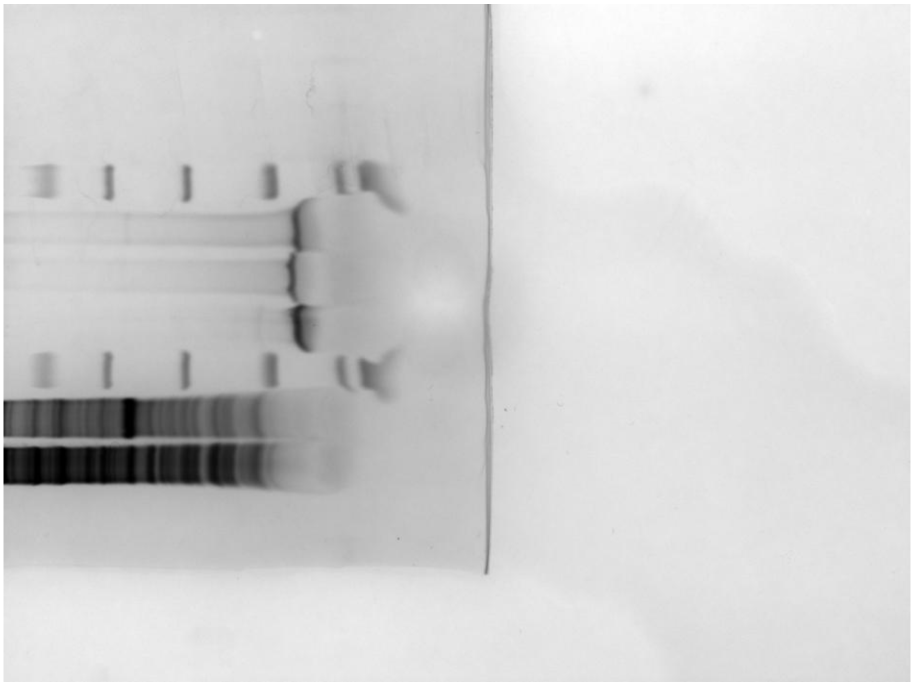

The original gel image used for the Supplementary Fig. 3c.
