# Supplementary material for: Direct detection of an NH-π hydrogen bond in an intrinsically disordered peptide
Source: Nat Commun. 2025 Nov 20;16:10231. doi: 10.1038/s41467-025-66013-2 (PMC12635323; doi:10.1038/s41467-025-66013-2)
Supplement: Supplementary file 2 — Reporting Summary [file 41467_2025_66013_MOESM2_ESM.pdf]

nature portfolio

Corresponding author(s): Nasrollah Rezaei-Ghaleh

Last updated by author(s): Oct 25, 2025

Reporting Summary

Nature Portfolio wishes to improve the reproducibility of the work that we publish. This form provides structure for consistency and transparency in reporting. For further information on Nature Portfolio policies, see our Editorial Policies and the Editorial Policy Checklist.

Statistics

For all statistical analyses, confirm that the following items are present in the figure legend, table legend, main text, or Methods section.

n/a	Confirmed	
☐	☒	The exact sample size (*n*) for each experimental group/condition, given as a discrete number and unit of measurement
☐	☒	A statement on whether measurements were taken from distinct samples or whether the same sample was measured repeatedly
☒	☐	The statistical test(s) used AND whether they are one- or two-sided<br>*Only common tests should be described solely by name; describe more complex techniques in the Methods section.*
☒	☐	A description of all covariates tested
☒	☐	A description of any assumptions or corrections, such as tests of normality and adjustment for multiple comparisons
☒	☐	A full description of the statistical parameters including central tendency (e.g. means) or other basic estimates (e.g. regression coefficient) AND variation (e.g. standard deviation) or associated estimates of uncertainty (e.g. confidence intervals)
☒	☐	For null hypothesis testing, the test statistic (e.g. *F*, *t*, *r*) with confidence intervals, effect sizes, degrees of freedom and *P* value noted<br>*Give P values as exact values whenever suitable.*
☒	☐	For Bayesian analysis, information on the choice of priors and Markov chain Monte Carlo settings
☒	☐	For hierarchical and complex designs, identification of the appropriate level for tests and full reporting of outcomes
☒	☐	Estimates of effect sizes (e.g. Cohen's *d*, Pearson's *r*), indicating how they were calculated

Our web collection on statistics for biologists contains articles on many of the points above.

Software and code

Policy information about availability of computer code

Data collection	NMR data collections were performed using Bruker TopSpin V 3.x or 4.x. NMR experiments were conducted using standard Bruker or previously published pulse sequences, as cited in the paper. The Bruker NMR pulse sequences and acquisition parameters (plus raw NMR data) have been deposited in the Zenodo database under accession code 17410262 (https://doi.org/10.5281/zenodo.17410262). The standard and refocused 13C,1H SOFAST-HMQC experiments were conducted using slightly modified pulse sequences, as described in the Methods and in the Caption to Supplementary Fig. 19. The linear peptide models were constructed using the Molecular Operating Environment (MOE) 2022.02. Protonation states were assigned according to PROPKA2, as cited in the paper. Molecular Dynamics (MD) simulations were performed using GROMACS software suite 2022.1. The essential input files used for the MD simulations have been deposited in the Zenodo database under accession code 16737783 (https://doi.org/10.5281/zenodo.16737783). DFT calculations were carried out using Gaussian 16 Rev.C.02. The input parameters used for DFT calculations (density functionals, basis sets, solvent models, optimized Cartesian coordinates) have been reported in the Methods or included in the Supplementary Information.

Data analysis	NMR data analyses were performed using Bruker TopSpin V 3.x or 4.x, NMRPipe V 12.2, CARA V 1.9.1.7 and NMRFARM-SPARKY V 1.2. Data fitting and plots were performed using OriginPro V 9.0. Random coil chemical shifts for Ha secondary chemical shift analysis were predicted, as cited in the paper, using the online javascript (https://www1.bio.ku.dk/english/research/bms/sbinlab/randomchemicalshifts2/).The MD trajectories were visualized and analyzed using VMD 1.8.6 and MDAnalysis 2.9.0 Python package for NH-pi interactions. The custom codes used in this study are deposited in the Zenodo database under accession code 16737783 (https://doi.org/10.5281/zenodo.16737783) or available in GitHub (https://github.com/davari-group/MD-Distance-Angle-Analysis or https://doi.org/10.5281/zenodo.17417064). The NCIPlot program was used for non-covalent interactions (NCI) analysis of the wavefunctions extracted from the Gaussian (Gaussian 16 Rev.C.02) calculations. The Gnuplot 4.4 program was used for the generation of NCI results. The Multiwfn program version 3.8 was used for Raman analysis of the extracted wavefunctions.

For manuscripts utilizing custom algorithms or software that are central to the research but not yet described in published literature, software must be made available to editors and reviewers. We strongly encourage code deposition in a community repository (e.g. GitHub). See the Nature Portfolio guidelines for submitting code & software for further information.

Data

Policy information about availability of data

All manuscripts must include a data availability statement. This statement should provide the following information, where applicable:

- Accession codes, unique identifiers, or web links for publicly available datasets
- A description of any restrictions on data availability
- For clinical datasets or third party data, please ensure that the statement adheres to our policy

Source data for Fig. 1c and Supplementary Figs. 2b,c, 5 and 6 are provided with the paper (in separate sheets of an Excel file). The molecular simulations generated in this study have been deposited in the Zenodo database under accession code 16737783 (https://doi.org/10.5281/zenodo.16737783). DFT optimized coordinates for the peptide models are given in the Supplementary information file as Supplementary Table 1. The raw NMR data (plus NMR acquisition parameters) have been deposited in the Zenodo database under accession code 17410262 (https://doi.org/10.5281/zenodo.17410262).
The custom codes used in this study are deposited in the Zenodo database under accession code 16737783 (https://doi.org/10.5281/zenodo.16737783) or available in GitHub (https://github.com/davari-group/MD-Distance-Angle-Analysis or https://doi.org/10.5281/zenodo.17417064).

Research involving human participants, their data, or biological material

Policy information about studies with human participants or human data. See also policy information about sex, gender (identity/presentation), and sexual orientation and race, ethnicity and racism.

Reporting on sex and gender	n/a
Reporting on race, ethnicity, or other socially relevant groupings	n/a
Population characteristics	n/a
Recruitment	n/a
Ethics oversight	n/a

Note that full information on the approval of the study protocol must also be provided in the manuscript.

Field-specific reporting

Please select the one below that is the best fit for your research. If you are not sure, read the appropriate sections before making your selection.

☒ Life sciences ☐ Behavioural & social sciences ☐ Ecological, evolutionary & environmental sciences

For a reference copy of the document with all sections, see nature.com/documents/nr-reporting-summary-flat.pdf

Life sciences study design

All studies must disclose on these points even when the disclosure is negative.

Sample size	NMR: samples were prepared freshly before each round of optimization or data production experiments which typically lasted 7-10 days. All the NMR data obtained through production experiments are included. The long-range 13C,1H SOFAST HMQC experiment (data shown in Fig. 4) was performed three times. Number of time points in the direct/indirect dimensions were adapted for resolution and signal to noise optimization. Number of relaxation data points were chosen to optimize the sampling of relaxation curves.<br>Raman: Raman spectra of each of the studied peptides (Supplementary Figs. 8 and 9) were collected from two samples, at 2 (the first sample) or 10 (the second sample) spots of the sample.<br>MD simulation: MD trajectories were run for three replicas of the studied peptides.
Data exclusions	No data were excluded.

Replication	NMR: reproducibility of NMR samples were checked through standard 1D 1H and 2D 1H,1H TOCSY experiments for the non-labeled peptides and 1D 1H and 2D 15N,1H HSQC experiments for the uniformly 15N-labeled peptides (and 1D 1H 1D 19F experiments for the 19F-labeled peptides). All attempts at replication were successful: the key observation in the NMR spectra of the E22G Abeta peptide, i.e. the upfield chemical shift of the amide proton of Gly22 and its near-zero temperature coefficient, was well reproduced in all the measured samples. The replication of J-based correlation through NH-pi hydrogen bond (shown in Fig. 4) was confirmed three times on the E22G Abeta sample. Raman: The reproducibility of the Raman spectra (Supplementary Figs. 8 and 9) was confirmed by two different Raman labs (Univ. Trento, Univ. Pavia), on two samples and at multiple spots of those samples.
Randomization	No randomization was performed. We have included all the relevant NMR and Raman spectroscopy and MD simulation and DFT data in our analysis, the randomization of data was not required.
Blinding	No blinding was performed. The present study did not involve any clinical trial, animal study, immunoassay or similar experiments. The blinding methods (single, double) were not applicable to this study.

Reporting for specific materials, systems and methods

We require information from authors about some types of materials, experimental systems and methods used in many studies. Here, indicate whether each material, system or method listed is relevant to your study. If you are not sure if a list item applies to your research, read the appropriate section before selecting a response.

Materials & experimental systems

n/a	Involved in the study
☒	☐ Antibodies
☒	☐ Eukaryotic cell lines
☒	☐ Palaeontology and archaeology
☒	☐ Animals and other organisms
☒	☐ Clinical data
☒	☐ Dual use research of concern
☒	☐ Plants

Methods

n/a	Involved in the study
☒	☐ ChIP-seq
☒	☐ Flow cytometry
☒	☐ MRI-based neuroimaging

Plants

Seed stocks	n/a
Novel plant genotypes	n/a
Authentication	n/a
